# Liquid organic hydrogen carriers for hydrogen storage: advances, challenges, and technoeconomic and sustainability perspectives

**DOI:** 10.1039/d6ra01860b

**Published:** 2026-08-12

**Authors:** M. Shahabuddin, Raza Moshwan, Md Rezaur Rahman, N. W. M. Zulkifli, Y. Y. Liang

**Affiliations:** a Department of Mechanical Engineering, Faculty of Engineering, University of Malaya 50603 Kuala Lumpur Malaysia shahabuddin@um.edu.my shahabuddin@iubat.edu; b Department of Mechanical Engineering, International University of Business Agriculture and Technology Dhaka 1230 Bangladesh; c Department of Chemical Engineering and Energy Sustainability, Universiti Malaysia Sarawak Malaysia; d Faculty of Chemical and Process Engineering Technology, Universiti Malaysia Pahang Al-Sultan Abdullah Lebuh Persiaran Tun Khalil Yaakob 26300 Kuantan Pahang Malaysia

## Abstract

Liquid Organic Hydrogen Carriers (LOHCs) have emerged as one of the most promising chemical-based options for hydrogen storage and transport. The review critically analysed peer-reviewed literature, primarily published recently, with emphasis on recent development, challenges and mitigating strategies. The review finds that Toluene-Methylcyclohexane (Toluene/MCH), benzyl toluenes (BT), dibenzyl toluenes (DBT), and *N*-ethylcarbazole (NEC) are technically advanced LOHC families with commercial applications. For hydrogenation reactions, noble metals, primarily Pt, Pd, and Ru, supported on oxide surfaces are widely used. The reaction temperature for the Toluene/MCH and NEC systems ranges from 180 to 225 °C, whereas BT and DBT require around 300 °C. For dehydrogenation, major catalyst/support systems include Pt/KIT-6 mesoporous silica, Pt/TiO_2_, Pt/Al_2_O3, Ru/Al_2_O_3_, Pt/V_2_O_5_, Pt/Y_2_O_3_, and Pt–Re/Al_2_O_3_. The dehydrogenation reaction critically suffers from a high-temperature requirement of ∼250–450 °C, due to its endothermic nature. Various types of reactors are used for the LOHC process, including fixed-bed, fluidised-bed, and Trickle/slurry-bed reactors. Significant challenges of LOHC systems include the high cost of catalysis, especially with noble catalysts; catalyst deactivation; high temperature/energy requirements, especially during dehydrogenation; carbon coking; slow reaction rates; instability under cycling; and by-product formation. From a technoeconomic perspective, the LOHC system is expensive, with a range of $2.0–7.0/kg_H_2__ depending on the carrier and catalyst used. Therefore, LOHC technology necessitates a comprehensive, system-wide strategy rather than molecular-level advancements. One such strategy might be efficient heat utilisation in low-carbon hydrogen logistics, including integrated methods such as microwave and exhaust heat recovery. The management of carriers must align with chemical properties and logistics, while system-level techno-economic analysis (TEA) and life cycle assessment (LCA) are essential. Innovations in catalysts focus on inductively heatable structures and durability during cycles, while reactor advancements aim to improve temperature regulation and address transmission constraints.

## Abstract 2

This review critically assesses LOHCs, integrating hydrogen storage fundamentals, kinetics, catalysts, reactor engineering, techno-economic, environmental sustainability, key challenges, and future research directions.

## Graphical abstract

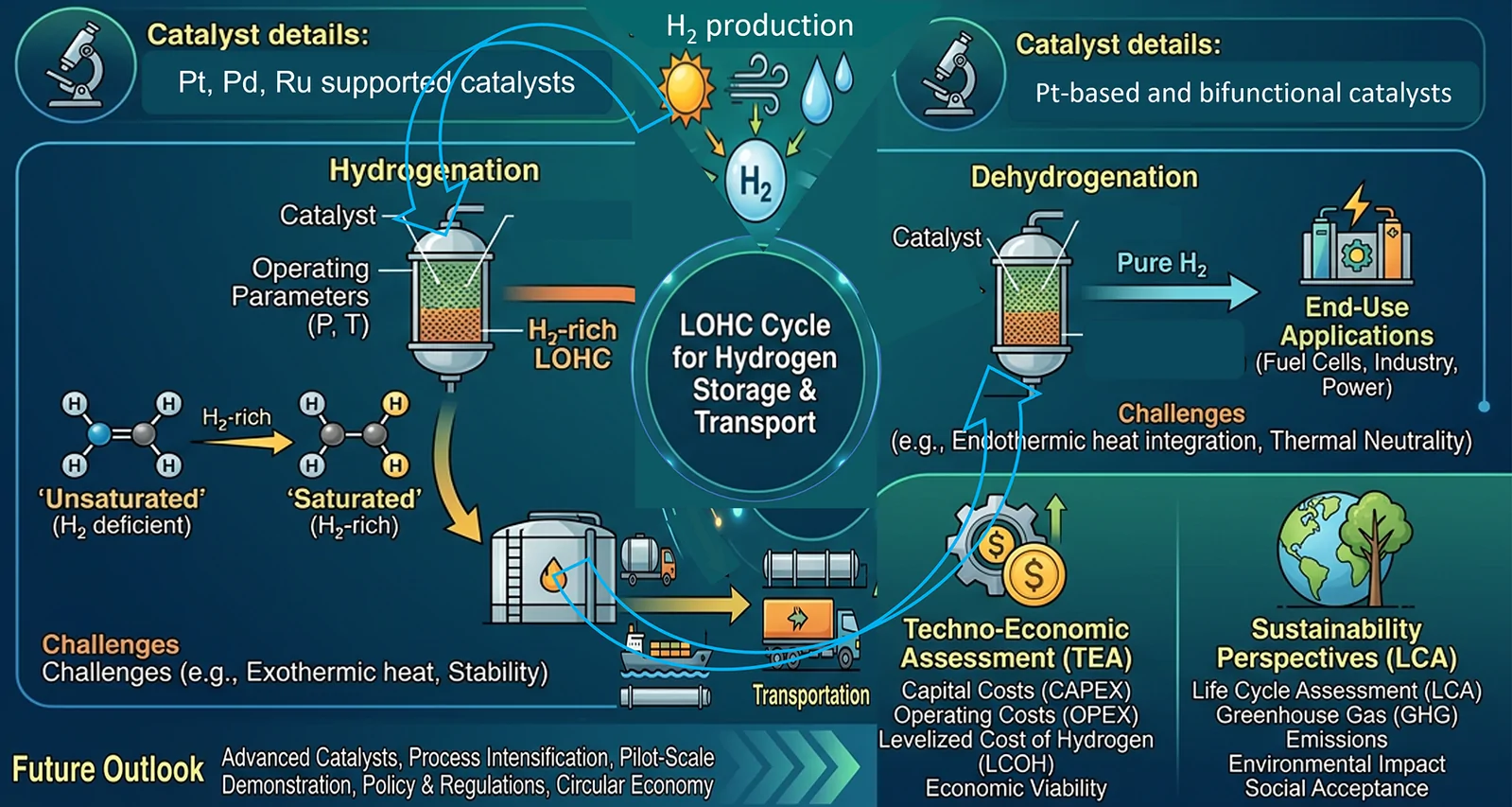

## 1. Introduction

One of the key challenges with hydrogen technology is its storage and transportation, particularly due to low volumetric energy density. The low volumetric density of hydrogen and its extreme storage conditions (cryogenic liquefaction or high-pressure compression) make large-scale, long-distance distribution difficult, thereby posing a key barrier to the development of the hydrogen economy.^1,2^ Hydrogen storage systems differ significantly when compared on a volumetric hydrogen density basis (kg_H_2__ m^−3^). In general, hydrogen can be stored as compressed hydrogen (CH_2_), liquefied hydrogen (LH_2_), in underground caverns, or bound in solid or liquid storage materials.^3^

Compressing hydrogen at 350 to 700 bars results in hydrogen densities of about 15–40 kg_H_2__ m^−3^. In contrast, liquid hydrogen offers a higher energy density of about 71 kg_H_2__ m^−3^, making it appealing for long-distance transportation. However, liquefaction of hydrogen requires extremely low temperatures and is considered highly energy-intensive.

Underground salt caverns typically store hydrogen at densities between 8 and 18 kg_H_2__ m^−3^, depending on operating pressure, offering the largest storage capacities with the lowest storage costs. With effective hydrogen storage densities usually between 47 and 60 kg_H_2__ m^−3^, liquid organic hydrogen carriers (LOHCs) are a promising alternative to liquification without the need for cryogenic temperatures. As such, LOHCs can store far more hydrogen per unit volume than underground caverns and compressed hydrogen. LOHCs are a viable choice and have attracted increasing attention in recent years, especially for medium- and long-distance hydrogen transportation and distribution.^4–6^ The distinct advantages of LOHCs include their high volumetric hydrogen density, the ability to store and transport hydrogen under ambient temperature and pressure, and compatibility with conventional liquid fuels such as diesel.

LOHCs store hydrogen by reversibly attaching hydrogen to an organic liquid through catalytic hydrogenation (H_2_ loading) and releasing it through catalytic dehydrogenation (H_2_ unloading).^7^ Unlike compressed or liquefied hydrogen, LOHCs often remain liquids at near-ambient conditions, enabling handling and transport similar to those of traditional liquid fuels and chemical commodities.^8^

Due to their liquid-fuel-like characteristics, LOHCs can improve reliability by reducing infrastructure barriers and utilising existing knowledge and components used for liquid logistics, such as tanks, pumps, pipelines, and tankers.^9^ Moreover, LOHC technology is relevant to the regional and transoceanic hydrogen supply chain, as it enables stable hydrogen storage and large-scale transportation.^10^ The LOHCs have the advantage of preserving hydrogen in a dense, manageable liquid medium and permitting on-demand release as high-purity gas when required for fuel cells, industrial operations, or synthesis. LOHCs can therefore serve as both a transport vector and a storage medium without the need for separate technologies.^9^

LOHCs, however, represent a range of process configurations and carrier chemistries, each with trade-offs. In the literature, three LOHC families have been used widely, including cycloalkane/aromatic (*e.g.*, toluene/methylcyclohexane), high-boiling aromatic (*e.g.*, the dibenzyltoluene/perhydrodibenzyltoluene family), and N-heterocyclic compounds (*e.g.*, *N*-ethylcarbazole).^11,12^ The ideal LOHC candidates are those that strike a practical balance among hydrogen capacity, chemical stability, safe handling, availability, and catalytic efficacy across repeated cycles.^10^ Various carrier classes of LOHCs differ considerably in handling qualities, thermodynamic requirements, and process integration demands.^9^ Hence, LOHC selection must be based on the application environment. In the literature, numerous organic compounds, including aromatic hydrocarbons (*e.g.*, toluene), nitrogen-containing heterocyclic compounds (*e.g.*, *N*-ethylcarbazole), and other carbazole derivatives, have been widely investigated as LOHCs.^13^ For instance, Muller *et al.*^14^ reported that, due to specific advantageous properties, such as high thermal stability and good hydrogen capacity, the dibenzyl toluene (H_0_-DBT)/perhydro dibenzyl toluene (H_18_-DBT) LOHC system is of special relevance for practical and industrial applications.

Hydrogen storage *via* physisorption typically requires low temperatures or high pressures to achieve appreciable storage capacities, whereas hydrogen chemisorbed in storage materials is generally released at elevated temperatures.^15,16^ The LOHC hydrogenation and dehydrogenation system comprises four key components: a chemical reactor, a hydrogen gas purification system, a heat integration system, and an electrochemical conversion system.^17^ Hydrogenation is generally exothermic and can often be driven effectively with an industrial catalytic reactor, whereas dehydrogenation is endothermic and can impose a substantial heat-supply and heat-transfer burden.^18^ Because LOHC dehydrogenation is highly endothermic, reactor performance is often limited by heat transfer rather than intrinsic reaction kinetics. Consequently, efficient heat management is essential to achieve and sustain high hydrogen production rates while maintaining the required operating temperature.^17^ This imbalance drives system-level design choices: heat integration, reactor selection (*i.e.*, fixed-bed, trickle-bed, slurry), and catalyst durability, all of which are decisive for techno-economic feasibility.^19^ As summarised in Table 1, hydrogenation is typically influenced by mass-transfer and kinetic limitations, whereas dehydrogenation is predominantly limited by heat transfer and catalyst stability.

**Table 1:** Comparative analysis of hydrogenation and dehydrogenation processes in LOHC systems^20–24^

Aspect	Hydrogenation	Dehydrogenation
Thermodynamics	Exothermic reaction	Endothermic reaction
Heat management	Heat removal required	Continuous heat supply required
Rate-limiting step	Mass transfer and catalyst wetting	Heat transfer and desorption kinetics
Catalyst systems	Pt, Pd, Ru supported catalysts	Pt-based and bifunctional catalysts
Catalysts challenges	Sintering and hydrogenation selectivity	Coking, deactivation, and poisoning
Scale-up challenges	Gas-liquid mass transfer limitations	Heat transfer limitations and energy demand

Furthermore, repeated LOHC charging and discharging cycles require efficient hydrogenation and dehydrogenation processes with precise control over hydrogen uptake and release. These processes must minimise undesirable reactions such as thermal cracking, polymerization, catalyst deactivation, and side-product formation, which can reduce hydrogen recovery and degrade the carrier material over time. Consequently, the development of highly active, selective, and durable catalysts remains a critical factor in improving the efficiency, stability, and long-term viability of LOHC systems.^25^ Jangir *et al.*^26^ reported that heterogeneous catalyst systems have demonstrated significant progress in reversible LOHC reactions, while emphasising that catalyst stability, catalyst selection, and support properties remain critical factors for enhancing long-term performance.

LOHC adoption must address energy integration, catalyst performance, and overall supply-chain issues.^9^ Furthermore, depending on the LOHC type and operating conditions, hydrogen purification may be required.^27^ These challenges are particularly important because LOHCs compete with a wide range of hydrogen storage technologies, including CH_2_, LH_2_, chemical hydrogen carriers (*e.g.*, ammonia and methanol), and solid-state storage materials. Therefore, LOHC advantages must be assessed in the context of overall system efficiency, operability, and sustainability.

Recent studies on LOHC predominantly emphasise materials design, catalytic mechanisms, and molecular modelling.^9,27^ Molecular design plays an important role in determining the properties and behavior of carriers.^28^ However, the performance of LOHC at the engineering scale depends on interactions among system design aspects related to operation, heat management, logistics, and process optimisation, indicating that molecular properties determine the performance limits. In contrast, system design determines the efficiency of their implementation in developing effective hydrogen transportation and storage technologies.

Previously conducted reviews of LOHCs were primarily focused on specific areas of the technology, such as the synthesis of carrier molecules, catalysts, reactors, or feasibility analysis. Nevertheless, there have been few attempts to consider the interplay among molecular properties, reactor constraints, heat management issues, durability, feasibility analysis, and life-cycle analysis in terms of implementation. The current review thus aims to take a systems approach, encompassing the selection of the carrier, reaction engineering constraints, durability, and the scaling of the process across the hydrogen supply chain. The main feature that distinguishes the current review from previous ones is its focus on the actual implementation of technologies. As such, (i) reviewing fundamental aspects of LOHCs and critically comparing leading LOHC carrier families through a system lens that links molecular properties to dehydrogenation heat demand and reactor constraints, and (ii) identifying advancement and actionable challenges, particularly around durability under realistic cycling, thermal neutrality strategies, application-specific carrier selection, technoeconomic and lifecycle analysis.

Although this review primarily focuses on LOHC technologies as a promising pathway for hydrogen storage and transportation, other hydrogen storage options, including CH_2_, LH_2_, metal hydrides, and chemical carriers such as ammonia, are briefly discussed for comparison purposes. The primary emphasis is placed on LOHC carrier families, hydrogenation and dehydrogenation processes, catalyst development, reactor design, techno-economic performance, and environmental impacts. The review critically analysed peer-reviewed literature published primarily over the last 5 years (>50%). Studies were selected based on their relevance to carrier properties, catalytic performance, process integration, system efficiency, economic feasibility, and sustainability, while studies lacking sufficient technical or performance data were excluded.

## 2. Fundamentals of hydrogen storage

**Fig. 1:**
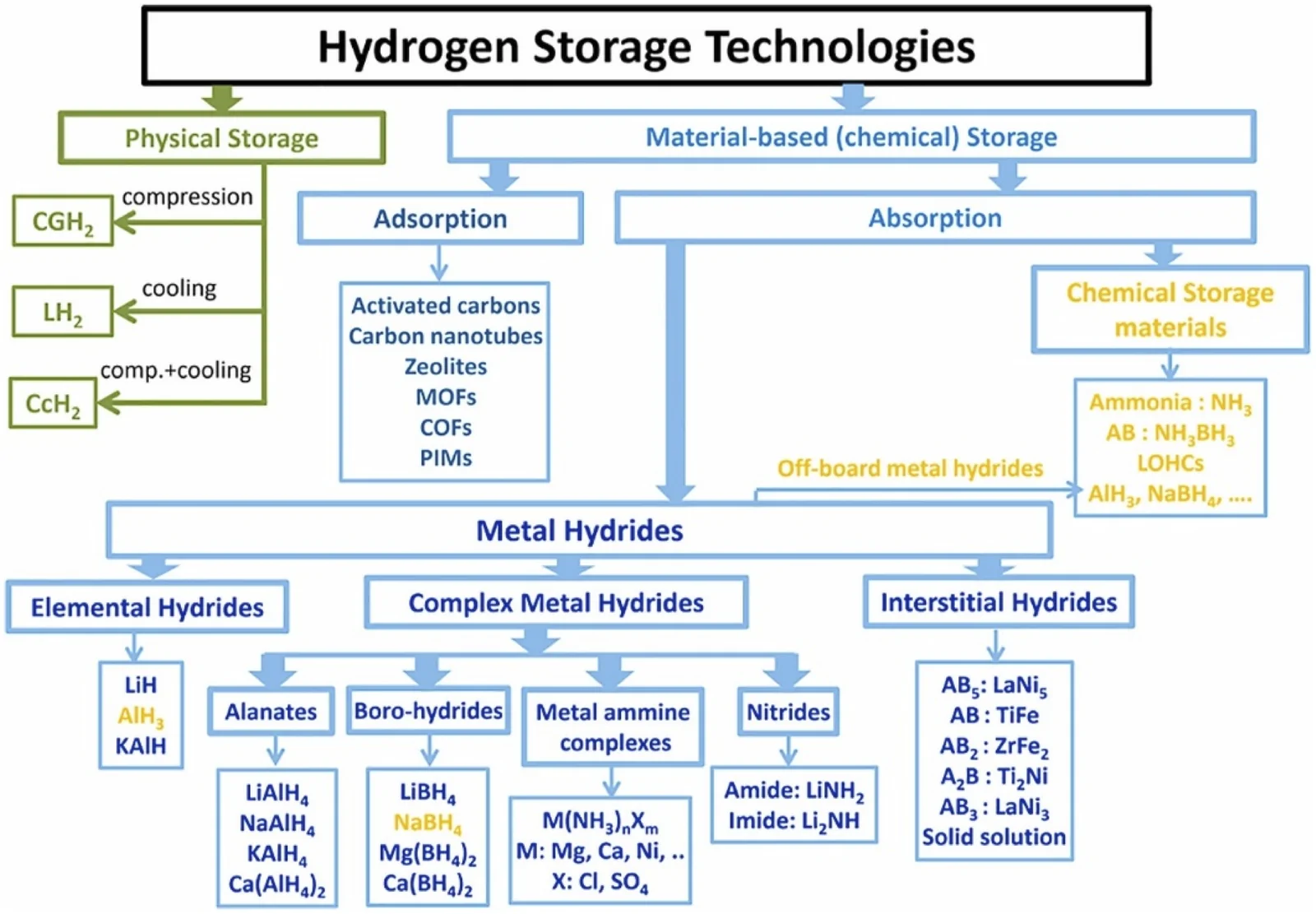
Hydrogen storage pathways, reproduced from ref. 33 with permission from Elsevier, copyright 2026.

The primary methods for hydrogen storage include physical, chemical, and material-based techniques, as shown in Fig. 1.^29^ Physical storage utilises compressed hydrogen gas at elevated pressures (350–700 bar) or liquid hydrogen at cryogenic temperatures (−253 °C).^30^ Chemical storage technologies encompass liquid organic hydrogen carriers (LOHCs), ammonia, and metal hydrides.^31^ LOHCs chemically bind hydrogen to organic compounds, such as methylcyclohexane or benzyl-toluene, which are safe to handle and compatible with the current liquid fuel infrastructure, though they require energy-intensive dehydrogenation procedures.^32^

Ammonia is considered a promising hydrogen carrier owing to its high hydrogen content of 17.6 wt%, making it an attractive option for hydrogen storage and transportation. However, its synthesis and decomposition consume substantial energy and pose a risk of toxicity. Although metal hydrides offer high volumetric density by bonding hydrogen to metallic alloys, their weight and expense limit their use in mobility applications. Adsorption on porous materials such as metal–organic frameworks (MOFs), carbon nanostructures, and cryo-adsorbed hydrogen storage are examples of material-centric storage techniques that represent cutting-edge technology with potential for lightweight and compact applications.^34^ In addition to classical porous nanostructured carbon materials and carbon-supported catalysis, chemically confined carbon architectures, particularly fullerene cages and endohedral fullerene systems, represent a promising new avenue for molecular design. In these spatially confined environments, embedded atoms are subjected to constrained carbon bonding that is substantially different from conventional hybridization models. This confinement permits novel bonding states, such as multicentre carbon-metal bonds, and reduces Coulomb repulsion, as recently reviewed in detail by Zhao *et al.*^35^ These endohedral and chemically confined systems provide new opportunities for the development of highly tunable next-generation carbon-based storage materials and catalysts.

**Fig. 2:**
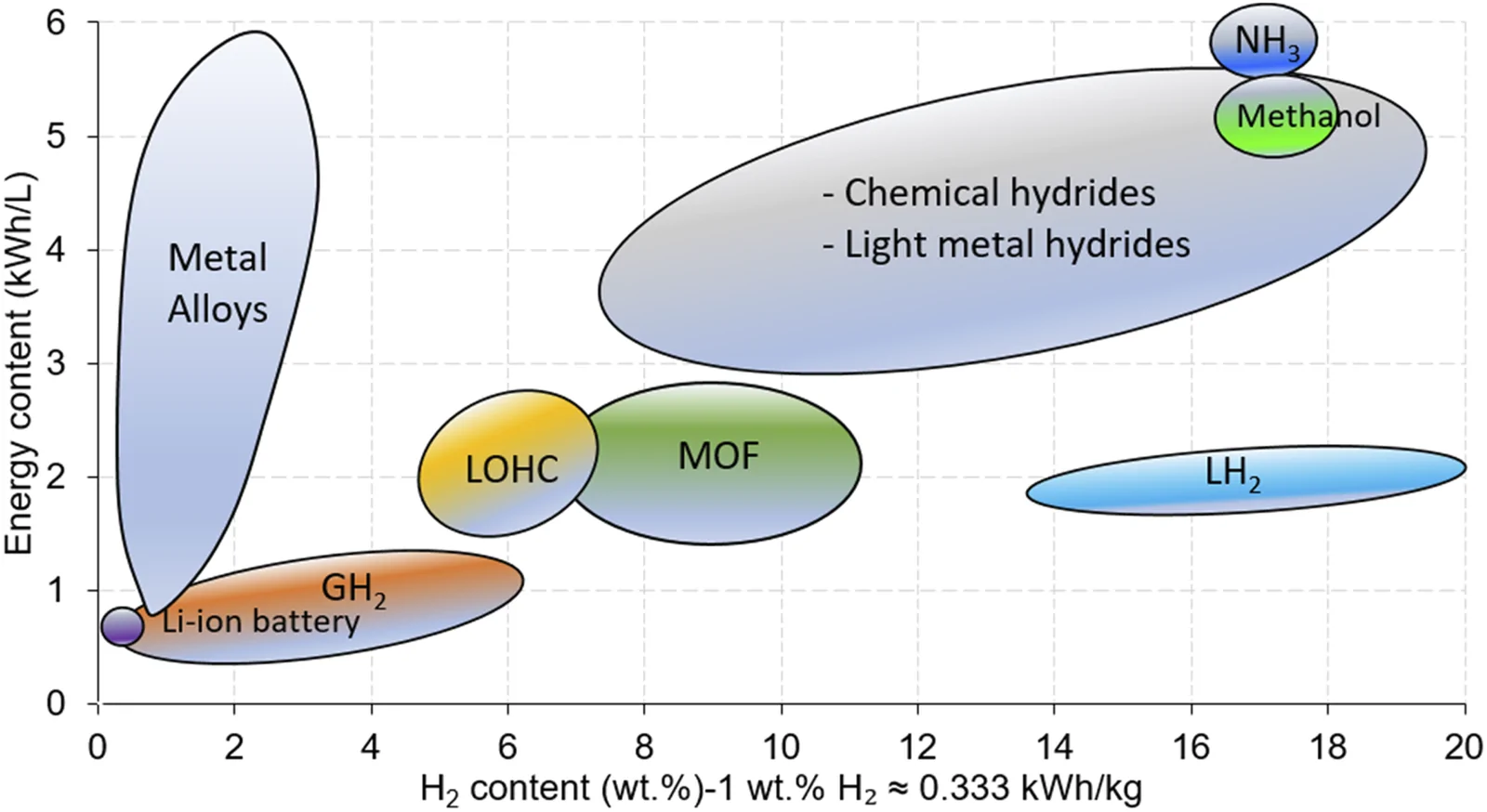
Comparative H2 content and corresponding energy content of LOHC with other hydrogen storage technologies, reproduced from ref. 19 and 37 with permission from Elsevier, copyright 2026.

Among such materials comprising carbon and porous components, advances in chemically confined carbon structures, particularly fullerene cages and endohedral fullerenes, expand the existing range of carbon-based nanostructures used in LOHC catalysts.^35,36^ Under such confinement, non-traditional bonding between carbon and metal, as well as the formation of highly charge-reduced systems, becomes possible, thereby allowing reactive intermediates to be stabilized with their specific electronic configurations. Nonetheless, many technologies remain in experimental phases, facing difficulties of scalability and affordability. Fig. 2 shows the variations in H_2_ capacity and corresponding energy content of LOHCs compared to other hydrogen storage technologies, such as MOFs, LH_2_, and Gaseous Hydrogen (GH_2_).

Every hydrogen storage system involves trade-offs among energy density, cost, safety, and maturity. Compressed and liquid hydrogen are the most mature technologies, while LOHCs and ammonia are increasingly used for large-scale and industrial applications. Systems based on metal hydrides and adsorption are being developed concurrently for specific uses. Notwithstanding these improvements, the market faces issues, including higher energy demands for storage operations, costly infrastructure, and the need to maintain safety under extreme temperatures and pressure. Intensive research and innovation are necessary to overcome these constraints and permit the extensive application of hydrogen storage technologies to promote a sustainable energy future.

Table 2 compares the key attributes of common hydrogen storage technologies in terms of storage capacity, operating conditions, costs and technology readiness level (TRL). Established physical storage methods, such as compressed and liquid hydrogen (TRL 8–9), deliver pure gas directly but incur high energy penalties for compression or liquefaction and require complex infrastructure. Chemical carriers such as ammonia and synthetic methanol have the highest volumetric capacities (>100 kg_H_2__ m^−3^), but require energy-intensive cracking or reforming and stringent purification to remove toxic or carbon-based by products. Solid-state options such as metal hydrides and adsorbents offer excellent safety and reversibility but suffer from low actual system capacities and high material costs at present. The Liquid Organic Hydrogen Carriers (LOHCs) fall in the intermediate zone. They enable safe, ambient storage using existing liquid-fuel infrastructure with moderate system capacities (∼5–7 wt%). Among listed LOHC candidates, while carriers such as naphthalene or benzene offer higher gravimetric capacities (7.2 wt% and above), they require intensive, expensive separation techniques to remove highly volatile or toxic vapours. Conversely, heavier, low-volatility carriers such as dibenzyl toluene (DBT) and benzyl toluene (BT) enable much simpler condensation and filtration steps to meet stringent fuel-cell purity standards.

**Table 2:** Key characteristics of common hydrogen storage technologies and the position of LOHC^16,32–34,38–47^

Hydrogen storage technology	Typical storage capacity (materials basis, wt%)	Actual system capacity (wt%)	Advantages	Limitations	Purification and application	Approximate levelized cost ($ per kg H_2_)	TRL
Compressed hydrogen (350–700 bar)	∼100, (15–40 kg_H_2__ m^−3^)	4–6	Mature technology, fast refuelling, simple system design	High compression energy (7–15% of H_2_ energy), heavy tanks, safety concerns due to high pressure	Fuel-cell vehicles, hydrogen refuelling stations, and short-term storage	0.15–0.40	9
Liquid hydrogen (LH_2_)	∼100% H_2_; (70.8 kg_H_2__ m^−3^)	7–15	Highest purity, very high volumetric density	Liquefaction energy (∼30–40% of H_2_ LHV), boil-off losses, cryogenic infrastructure	Aerospace, long-distance transport, and large-scale storage	1.5–4.3	8–9
Metal hydrides (*e.g.*, LaNi_5_, MgH_2_)	1–7.6; (50–150 kg_H_2__ m^−3^)	1–4	High volumetric density, excellent safety, and reversible storage	Heavy materials, slow kinetics, thermal management required, expensive alloys	Stationary storage, backup power, portable systems	0.5–2.5	5–8
Adsorbent materials (MOFs, activated carbon, CNTs)	∼4–8; (cryogenic); <2% at ambient conditions	1–3	Fast adsorption/desorption, lightweight, reversible	Low ambient-temperature capacity often requires cryogenic operation, high material cost	Portable storage, research applications	2.0–4.0	3–6
Liquid organic hydrogen carriers (LOHCs)	5–7%; (50–60 kg_H_2__ m^−3^)	4.5–6	Ambient storage and transport, existing liquid-fuel infrastructure, high safety	Dehydrogenation requires catalysts and heat; hydrogen purification may be needed, with an energy penalty	Large-scale transport, seasonal storage, and distributed hydrogen supply	1.5–3.0	5–7
Dibenzyltoluene (DBT/H18-DBT)	6.2%; (57 kg_H_2__ m^−3^)	—		—	Low vapor pressure allows simple condensation; trace VOCs are easily removed *via* carbon filters	—	—
Benzyltoluene (BT/H12-BT)	6.2%; (54 kg_H_2__ m^−3^)				Similar to DBT, low volatility makes separation easier, but high thermal stability limits the formation of side-product gases		
Toluene/Methylcyclohexane (MCH)	6.2%; (47 kg_H_2__ m^−3^)				High vapor pressure requires deep cooling and intense pressure swing adsorption (PSA) to meet fuel cell limits		
*N*-Ethylcarbazole (NEC/12H-NEC)	5.8%; (54 kg_H_2__ m^−3^)				Very low volatility; highly pure H_2_, but requires scrubbing for trace nitrogen/amine degradation products		
Naphthalene/Decalin	7.3%; (65 kg_H_2__ m^−3^)				High capacity, but thermal cracking during release can produce trace amounts of methane, requiring additional purification		
Benzene/Cyclohexane	7.2%; (56 kg_H_2__ m^−3^)				High capacity, but benzene's extreme toxicity and high volatility require extensive, expensive purification efforts		
Ammonia (NH_3_)	17.6; (∼108 kg_H_2__ m^−3^)	13–15	High hydrogen density, mature transport infrastructure, and low liquefaction pressure	Toxicity, cracking required before use, possible NO_*x*_ formation	International hydrogen transport, fertilizer and energy sectors	3.0–4.0	8–9
Synthetic fuels (*e.g.*, methanol)	Methanol: 12.5; (∼100 kg_H_2__ m^−3^)	10–11	Liquid at ambient conditions, existing infrastructure, and relatively easy handling	CO_2_ emissions if fossil-derived, reforming required, CO purification needed	Fuel distribution, marine transport, chemical feedstock	2.0–2.5	7–9

## 3. Fundamentals and classification of LOHCs

**Fig. 3:**
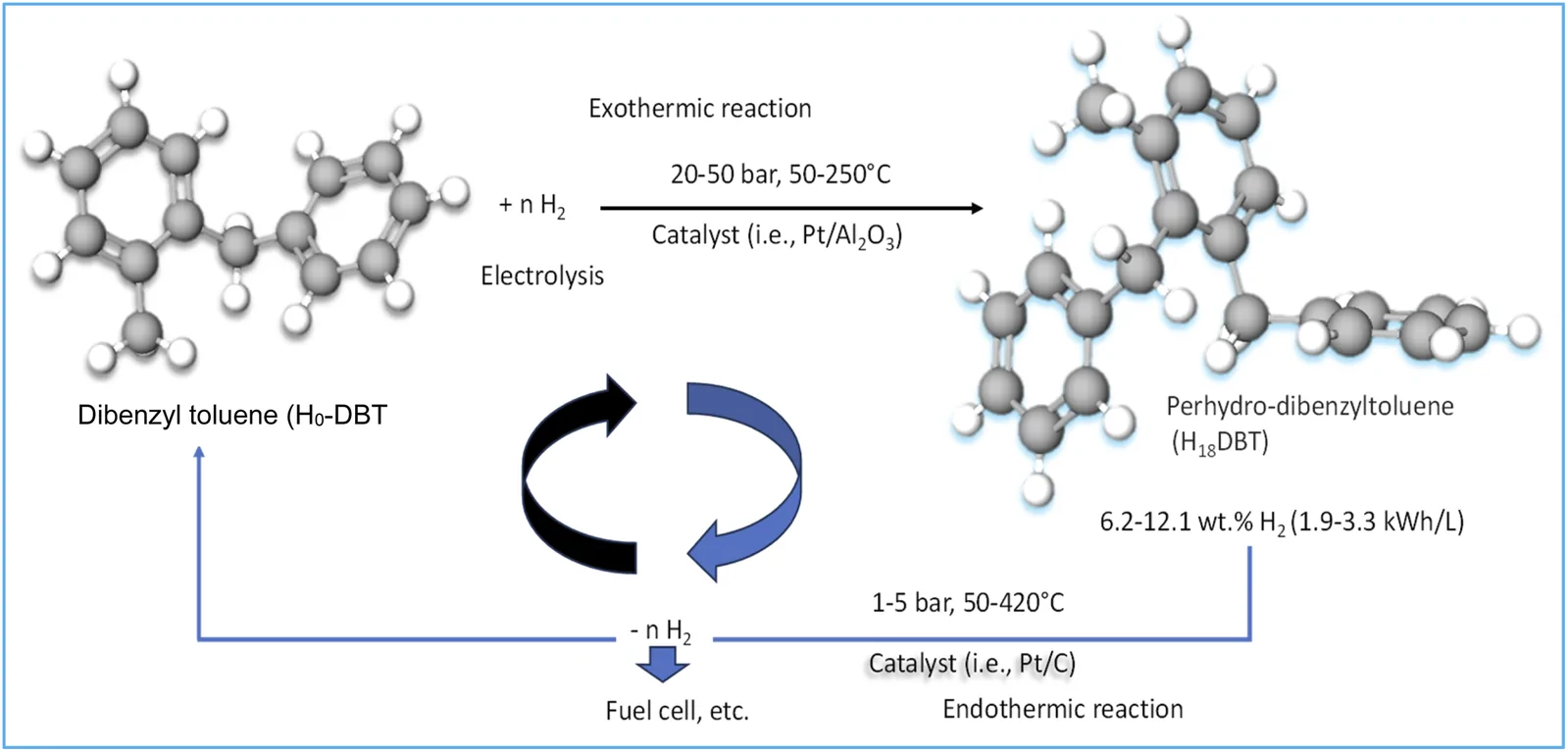
Schematic illustration of the LOHC concept with hydrogenation and dehydrogenation process, reproduced from ref. 47 with permission from Elsevier, copyright 2026.

LOHCs are liquids or low-melting solids that can be reversibly hydrogenated and dehydrogenated at elevated temperatures with the presence of a catalyst.^3^ The initial structure of the LOHC molecules remains the same when the rechargeable hydrogen is released. Fig. 3 depicts the LOHC cycle for the storage and transportation of hydrogen *via* reversible chemical processes, known as hydrogenation and dehydrogenation. During the hydrogenation phase, hydrogen interacts with the LOHC in an exothermic catalytic reaction, producing hydrogen-enriched H_*x*_-LOHC for storage and transportation.^11^ In the subsequent dehydrogenation process, heat is supplied in an endothermic catalytic reaction to release hydrogen and regenerate the original LOHC for reuse.^3,11^ A detailed literature review concerning these processes is presented in the next section.

The hydrogen emitted from LOHCs is quite pure. High selectivity at sufficiently mild reaction conditions is the goal of catalyst design to prevent covalent carbon–carbon bonds (or bonds with heteroatoms) from breaking, and the resulting cracking and coking.^48,49^ Some chemical structures are more susceptible to hydrogenation or dehydrogenation than others. Several studies in the literature reported the physicochemical properties of LOHC materials,^14,50–52^ phase equilibria^53–55^ and the environmental and health impact.^5^ Numerous studies have addressed the efficiency of LOHC-based energy storage^56–58^ and concepts for the application of the LOHC technology.^4,59^ Thus, this review deliberately skips a detailed discussion of those aspects.

A critical component of LOHC development is the systematic classification and rigorous selection of suitable carrier molecules. The literature typically categorises LOHC media according to molecular structure into homocyclic aromatic systems (aromatic rings composed solely of carbon atoms, such as benzene, toluene, or dibenzyltoluene) and heterocyclic or substituted aromatic systems, which incorporate heteroatoms (*e.g.*, N, O, S) or functional substituents within the ring or side chains.^46^ Homocyclic aromatic carriers exemplify early LOHC concepts, in which simple aromatics serve as the hydrogen-lean form (LOHC^−^) and their saturated cycloalkanes as the hydrogen-rich form (LOHC^+^), upon hydrogenation.^10^ Heterocyclic systems, including *N*-ethylcarbazole and other nitrogen-containing compounds, have attracted attention due to higher hydrogen capacities and tunable reaction thermodynamics afforded by heteroatoms, though they often require more sophisticated catalysts and precise reaction control.^60^ However, a recent investigation explores cyclic LOHCs, focusing exclusively on homocyclic and heterocyclic families.^61^

Müller *et al.*^14^ studied thermophysical properties of Benzyltoluene (BT) and Dibenzyltoluene (DBT). The benefit of DBT over BT is its low vapour pressure and higher boiling point (DBT 390 °C, BT 280 °C), while a lower viscosity of BT than that of DBT eases its handling (BT 2.6 mm^2^ s^−1^ and DBT 16.5 mm^2^ s^−1^ at 40 °C). Viscosity of hydrogenated H18-DBT is as high as 82.6 mm^2^ s^−1^ at 40 °C. The enthalpies of hydrogenation and dehydrogenation of BT are at a reasonable range, from 62 to 71 kJ mol^−1^ H_2_. Müller *et al.*^14^ found that the respective enthalpy is very similar for BT (−63.5 kJ mol_H_2__^−1^) and DBT (−65.4 kJ mol_H_2__^−1^).

Heterocyclic carriers, particularly *N*-ethylcarbazole (NEC) and its derivatives, such as dodecahydro-NEC (12H-NEC), constitute another primary class of LOHC candidates. The incorporation of nitrogen into the aromatic structure reduces the enthalpy of dehydrogenation relative to pure hydrocarbons, enabling hydrogen release at slightly lower temperatures and enabling more favourable kinetics under specific catalytic systems. Extensive studies have analysed NEC's catalytic pathways, demonstrating its viability for repeated cycling while highlighting that catalyst performance and stability under cyclic stress are critical determinants of system viability.^27^

For comparison, the enthalpy of hydrogenation for the *N*-ethylcarbazole (NEC) is from −50 to −53 kJ mol_H_2__^−1^. The hydrogenation of BT and DBT is enthalpically favoured over NEC, while hydrogen release from NEC is more favourable than from BT and DBT. The melting point of DBT is −48 °C, and the melting point of its fully hydrogenated form is even lower (−58 °C). The density of DBT (1.045 g cm^−3^) is more than approximately 13% higher than the density of the respective hydrogenated form (0.921 g cm^−3^), as the aromatic rings are planar while the hydrogenated rings are non-planar. The dynamic viscosity of hydrogenated BT is slightly higher than that of its dehydrogenated form. The surface tension of hydrogenated DBT is lower than that of the dehydrogenated form.^14^

When three aromatic rings of DBT are hydrogenated, six different hydrogenated forms may be present: H0, H6 (outer or middle ring), H12 (outer and middle ring or two outer rings) and H18 form. All of these structures have regioisomers (positions of the benzyl groups relative to the methyl group on the middle ring) and diastereomers (chiral carbon atoms).^14,62^ Consequently, determining the hydrogenation state of DBT is challenging with many analytical methods.

Aslam *et al.*^63^ optimised a reversed-phase high-performance liquid chromatography method for the separation and purification of four partially hydrogenated fractions: H0-DBT, H6-DBT, H12-DBT and H18-DBT. The method used a column of phenylhexyl silica as the stationary phase and 4 vol% water with 96 vol% acetone as the mobile phase. Müller *et al.*^64^ explored possibilities to define the degree of DBT hydrogenation using density, viscosity, refractive index and Raman spectroscopy. The most reliable correlations were found for density or refractive index. Uncertainty is higher for the correlation based on refractive index, which decreases by about 7% during hydrogenation, than for the density correlation, which decreases by about 13%. Inhetveen *et al.*^65^ found that Raman spectroscopy is a suitable method for determining the hydrogenation rate of DBT. The setup could be automated to take samples during the manufacturing process.

Do *et al.*^62^ used nuclear magnetic resonance (NMR) to determine the degree of hydrogenation of DBT. Chemical shifts in NMR spectra shift from the aromatic to the aliphatic region as hydrogenation increases. Other characteristics important for DBT process development have also been studied, such as the physical solubility of hydrogen in DBT^53^ and binary diffusion coefficients over a temperature range from −9 to 298 °C.^52^

Effective screening criteria for LOHC candidates extend beyond classification. A fundamental requirement is a high reversible hydrogen storage capacity, typically over ∼5 wt%, to be competitive with other storage methods.^39^ Thermodynamic and kinetic considerations are equally critical. Another selection dimension involves safety and environmental performance as elaborated in Section 8. Despite substantial progress, no single LOHC has yet met all ideal criteria simultaneously, reflecting a critical research gap. Table 3 outlines the selection criteria and recommended targets for the LOHC system based on the literature review. As seen, the selection criteria imply that feasible LOHC systems must meet several thermodynamic, kinetic, and physical property constraints simultaneously, and the trade-off between hydrogen capacity, reaction temperature, and transportation determines the system's feasibility.

**Table 3:** Selection criteria and recommended targets for LOHC Systems

Category	Selection parameter	Recommended target	Justifications	References
Physical properties	Melting point	<30 °C	Avoid solidification; no solvent addition	66
	Boiling point	>300 °C	Low vapour pressure enables easier H_2_ purification	46
	Viscosity	Low dynamic viscosity	Facilitates pumping and infrastructure compatibility	46
Storage capacity	Gravimetric capacity	> 6 wt% (≥5 wt% at tank system level)	Competitive hydrogen density	11, 66 and 67
	Volumetric capacity	>56 kg_H_2__ m^−3^	Comparable to compressed hydrogen storage	66
Energy density	Volumetric energy density	>1.6 MWh m^−3^ (LHV basis)	Competitive with liquid fuels	11 and 67
Thermodynamics	Hydrogen binding enthalpy	30–50 kJ mol_H_2__^−1^ (42–54 kJ mol^−1^ acceptable range)	Enables dehydrogenation < 200 °C at ∼1 bar	66
Energy efficiency	Dehydrogenation energy demand	≤ 10 kWh per kg H_2_ (thermal)	Techno-economic competitiveness	11 and 67
Reaction kinetics	Dehydrogenation rate	>3 g_H_2__ g^−1^ catalyst/min	Industrially viable hydrogen release rate	11 and 67
Selectivity	Conversion/selectivity	>90% conversion; > 99.8% selectivity	Minimise side reactions and degradation	11 and 67
Durability	Performance degradation	<0.01% per cycle	Long service life; reduced replacement cost	11 and 67
Compatibility	Impurity tolerance	Up to 20% impurities in H_2_ feed	Compatible with mixed gas hydrogenation streams	11 and 67
Economics	Production cost	Low-cost, scalable synthesis	Essential for large-scale deployment	46
Infrastructure	Logistics compatibility	Compatible with existing fuel infrastructure	Reduces CAPEX	66
Safety & environment	Toxicity	Non-toxic, non-hazardous	Must comply with transport and environmental regulations	46

## 4. Hydrogenation and dehydrogenation process

### 4.1 Hydrogenation process

LOHC technology relies on the reversible hydrogenation of an unsaturated liquid organic molecule and subsequent dehydrogenation to store and release molecular hydrogen, respectively. This reversible catalytic process enables hydrogen to be chemically bound and transported under relatively benign temperature and pressure conditions without the safety and volumetric penalties associated with compressed or cryogenic hydrogen storage alternatives.^10^

The hydrogenation step in LOHC systems is critical for efficient hydrogen storage, as it involves the exothermic catalytic addition of H_2_ to an unsaturated organic carrier (LOHC^−^) to form its hydrogenated counterpart (LOHC^+^). The effectiveness of this process depends on selecting robust catalysts and optimised reactor configurations that achieve high conversion and selectivity under practical operating conditions,^68^ which are highlighted in detail in the following section.

### 4.2 Operating parameters for hydrogenation

The hydrogenation reaction is typically carried out at elevated pressures (30–50 bar) to shift the equilibrium toward the hydrogenated product and at 150–250 °C to ensure adequate reaction rates without excessive energy consumption.^69^ These conditions reflect a trade-off between kinetics and thermodynamics, where higher pressures favour hydrogen uptake but increase capital and operational costs. In addition, reactor design significantly influences conversion efficiency and heat management.^70,71^ Fixed-bed reactors are widely employed for pilot and industrial-scale operations due to their simplicity and ease of catalyst loading.^27^ Trickle-bed reactors, in which liquid LOHC and gaseous H_2_ flow concurrently over a catalyst bed, can enhance gas–liquid contact and mass transfer, thereby boosting reaction rates.^72^ Recently, Lippert *et al.*^72^ studied continuous hydrogenation of benzyltoluene (H_0_-BT) in a trickle-bed reactor, focusing on process parameters such as hydrogen partial pressure, temperature, and feed degree. Key findings include the need for initial catalyst bed flooding to boost activity, minimal productivity increases with pressure (only 50% when raised from 5 to 30 bar), and a strong temperature relationship: increases from 160 °C to 200 °C led to a 2.7-fold increase in hydrogenation. In terms of degree of hydrogenation (DoH), nearly complete hydrogenation (95% DoH) was achieved at just 5.0 bar total pressure from a partially hydrogenated feed (61.3% DoH). Overall, H_0_-BT hydrogenation is primarily limited by mass transfer, facilitating efficient hydrogen storage at low pressures. In contrast, slurry reactors, in which catalyst particles are suspended in the liquid phase, offer superior heat distribution but pose challenges for catalyst recovery and separation.^73^

Heat integration is another crucial factor, as hydrogenation is exothermic; effective heat-removal strategies are necessary to avoid overheating key areas, which can degrade catalysts or favour side reactions. Coupling hydrogenation with downstream heat recovery systems can improve overall energy efficiency by using the released thermal energy in other process units or to assist dehydrogenation steps.^74^Table 4 summarises the hydrogenation process using common LOHC candidates, including catalyst types, reactor configurations, and performance. As shown in this table, hydrogenation for LOHC technology is relatively advanced, particularly with the use of noble-metal catalysts and proven reactors, which enable high reactivity at moderate pressures. However, there is no universal standard when measuring and comparing performance levels due to various parameters, such as differing catalyst loadings and reactor sizes.

**Table 4:** Overview of hydrogenation process using common LOHC candidates concerning catalyst types, reactor configurations, kinetics and performance

LOHC system	Typical catalyst (s)	Reactor configuration	Operating conditions	Kinetic characteristics	Advantages	Limitations	Ref.
Toluene to methylcyclohexane (MCH)	Noble metals (Pt, Pd, Ru) on oxide supports	Fixed-bed/trickle-bed (gas-liquid-solid)	185–225 °C; 0.3–2 MPa	Fast hydrogenation kinetics; activation energy ≈ 35–50 kJ mol^−1^, depending on the catalyst; equilibrium-limited at high conversion	Mature chemistry; good logistics compatibility; fast hydrogenation kinetics	Catalyst cost: heat removal needed (exothermic); overall system limited by dehydrogenation step	75–78
Dibenzyltoluene (DBT) to Perhydro-DBT (H18-DBT)	Mainly Ru-based and Pt/Pd catalysts (supported)	Fixed bed; continuous flow systems	120–250 °C; 30–50 bar	Activation energy ≈ 45–60 kJ mol^−1^; reaction often limited by heat and mass transfer due to high viscosity	DBT is low-volatility and stable, attractive for large-scale storage logistics	Viscosity/mass transfer constraints; concerns with catalyst deactivation; hydrogenation heat management	46 and 79
				Enthalphy: −65.4 kJ mol_H_2__^−1^			
*N*-ethylcarbazole (NEC) to 12H-NEC	Mainly Ru, also Pt/Pd, depending on design	Fixed-bed and batch screening reactors	Temperature: 130–180 °C and pressure 50–80 bar	Activation energy ≈ 30 to 70 kJ mol^−1^	High hydrogen capacity class; potentially lower energy requirement for dehydrogenation *vs.* hydrocarbons	Carrier/catalyst sensitivity; possible by-product formation; careful purity control needed	80 and 81
				Enthaplhy: −50 kJ mol_H_2__^−1^			
Hydrogenation of LOHCs	Noble metals are dominant; Ni-based materials are explored as a lower-cost alternative	Trickle-bed/slurry/fixed-bed	Conditions tuned by carrier and catalyst		Strong industrial knowledge base from refinery hydrogenation; scalable reactor choices	Non-noble catalysts may show lower activity or stability; long-term cycling is critical	11, 82 and 83
Hydrogenation process design	Catalyst choice depends on heat removal, selectivity, and stability	Heat-integrated fixed-bed and process loops	Exothermic heat must be controlled		Hydrogenation heat can be recovered for system efficiency	Hotspots can accelerate deactivation; scale-up requires thermal control and gas–liquid mass transfer optimisation	84–88

### 4.3 Dehydrogenation process

Dehydrogenation is a rate and efficiency-limiting step in most LOHC chains because it is intrinsically endothermic, strongly coupled to heat and mass transfer, and highly sensitive to catalyst stability and carrier purity. In practical terms, the viability of an LOHC pathway is often determined less by hydrogenation than by whether hydrogen can be released quickly and cleanly, with an acceptable energy penalty at the point of demand.

A significant distinction among LOHC candidates is that molecule structure influences both thermodynamics and the operational parameters. Cycloalkane-based systems (*e.g.*, MCH to toluene) are attractive for logistics and maturity, but typically require high-temperature dehydrogenation, making heat supply and catalyst durability key concerns.^27^ In contrast, dibenzyltoluene-based systems (H_18_-DBT to H_0_-DBT) benefit from favourable handling properties (as industrial heat-transfer-oil fluids), but dehydrogenation remains heat-intensive and is often studied at elevated temperatures, where side reactions and by-product formation can increase.^89^

N-Heterocycles (*e.g.*, NEC) are commonly utilised due to their ability to undergo (de)hydrogenation at significantly lower temperatures than numerous hydrocarbons. Most LOHCs of this class dehydrogenate at temperatures around 200 °C, while underscoring the critical importance of catalyst design and stability.^68^ The fundamental implication is that NEC-type systems shift the challenge from providing substantial heat at elevated temperatures to preserving selectivity and catalyst longevity while ensuring high hydrogen purity on a more chemically functionalised carrier. Nevertheless, N-heterocyclic LOHCs face challenges related to thermal stability, catalyst durability, and chemical degradation, despite offering more favourable dehydrogenation temperatures than hydrocarbon-based LOHCs.^68^

#### 4.3.1 Reactor configuration for dehydrogenation

Reactor configuration strongly influences dehydrogenation performance because hydrogen evolution within the liquid phase alters gas–liquid contact, reduces catalyst wetting, and limits heat transfer efficiency.^74^ Process intensification approaches are therefore gaining momentum. For example, a study comparing a stirred-tank reactor with a continuous-flow microchannel reactor for H_18_-DBT dehydrogenation reports an improved hydrogen production fraction in the microchannel configuration, consistent with intensified heat/mass transfer in small channels and better control of temperature gradients.^90^ Inductively heatable Pt-based catalyst materials for H_18_-DBT exhibit another intensification pathway: delivering heat more directly where the reaction occurs, potentially reducing thermal inertia and mitigating hotspots associated with external heating.^91^ From a modelling and scale-up standpoint, recent work on the continuous dehydrogenation of perhydro-DBT highlights the engineering relevance of liquid-phase operation and the need for robust models that couple kinetics with reactor transport effects.^92^

#### 4.3.2 Heat integration and thermal neutrality

Due to the significant heat requirement for dehydrogenation, system analyses are increasingly concentrating on thermal-neutrality strategies, specifically methods to provide heat without compromising net efficiency. A review,^93^ dedicated to application-oriented thermal neutrality for LOHC, discusses strategies such as microwave intensification, exhaust heat circulation, and even direct LOHC fuel cell concepts to reduce or avoid external heat demand. In contrast, another study^66^ presents realistic configurations in which a portion of the released hydrogen may be combusted to provide dehydrogenation heat, highlighting that, without careful integration, the dehydrogenation process can deplete excessive amounts of the stored energy.

A single parameter, such as a catalyst, reactor, or heat integration, cannot solve dehydrogenation challenges. The most convincing pathway is co-design, like selecting a carrier whose temperature window matches available heat sources, pairing it with catalysts validated under realistic impurity conditions, and deploying reactors that manage gas release while delivering heat efficiently.^10^Table 5 summarises the dehydrogenation process using common LOHC candidates, including catalyst types, reactor configurations, and performance. As shown in the table, the dehydrogenation stage appears to be the most challenging in LOHC processes, mainly due to high endothermic heat requirements, elevated reaction temperatures, and sensitivity to catalyst architecture and support materials. Ideal LOHC molecules should exhibit favourable enthalpies of hydrogenation/dehydrogenation so that the hydrogen release step is not prohibitively endothermic, and reaction kinetics that allow reasonable rates at practical temperatures without excessive heat input.^60^ Further, LOHC molecules must possess liquid-phase stability over multiple hydrogenation cycles, low volatility to prevent carrier loss, and compatibility with existing industrial infrastructure to facilitate adoption.^10^

**Table 5:** Overview of dehydrogenation process using common LOHC candidates concerning catalyst types, reactor configurations, kinetics and performance

LOHC system	Catalyst/support	Typical operating conditions	Kinetic characteristics	Advantages	Limitations	Ref.
Methylcyclohexane (MCH)	Pt/KIT-6 mesoporous silica	-Temperature of 250–450 °C	Activation energy ≈ 80–170 kJ mol^−1^ (dehydrogenation), depending on the catalyst	- Enhanced hydrogen release compared with Pt/SiO_2_ or Pt/Al_2_O_3_ due to improved dispersion and pore structure, facilitating gas release and mass transfer	Requires high dehydrogenation temperature; carbon coking observed over long runs	11, 77, 78, 94 and 95
	- Pt/TiO_2_, Pt/Al_2_O, Pt/V_2_O_5_, Pt/Y_2_O_3_, and Pt Re/Al_2_O_3_			- High H_2_ selectivity, activity and improved stability compared to other Pt supports		
Perhydro-dibenzyltoluene (H_18_-DBT)	Pt or Ru/Al_2_O_3_	Temperatures of 280–320 °C and pressure of 0.1–0.5 MPa	Activation energy ≈ 120–170 kJ mol^−1^	- Well-studied, reproducible catalyst system	- Side product formation	19
				- High thermal stability and low toxicity, and high energy density of 1.9 kWh L^−1^ (storage capacity 6.2 wt%)	- Expensive noble metal	11, 96 and 97
	Pt/KIT-6		Enthalphy: + 65.4 kJ mol_H_2__	- Higher H_2_ conversion and selectivity; stable structure	- High temperature demand	11, 94 and^98^
				- Pt/KIT-6 catalysts enhance dehydrogenation performance	- High dehydrogenation energy requirements (65 kJ mol_H_2__^−1^), and slow reaction rate	
				- Confined Pt in ordered pore silica increases residence time and boosts conversion relative to amorphous supports	- Complexity of support synthesis	
H18-DBT/Other metal surfaces	Pd subsurface & Pt Pd alloys	—	—	- Provides understanding for the design of improved catalysts	- Pd/Pt surface configurations affect LOHC adsorption/dehydrogenation	19 and 46
					- Environmentally hazardous	
					- Requires extensive experimental validation	
N-Heterocycles (*e.g.*, H0-NEC, 12H-NEC)	Noble metals (Pt, Pd, Ru) & bimetallic	Temperatures of 280–320 °C		- Lower dehydrogenation temperature potential	- Catalyst cost; sensitivity to carrier impurities	11 and 99–101
				- Established catalysts	- Slower kinetics than hydrogenation	
*N*-Heterocycles (*e.g.*, 12H-NEC)	PdCu/reduced graphene oxide nanoparticles	Temperatures of 280–320 °C	Activation energy 55–75 kJ mol	- Tunable activity	- Requires careful synthesis	11, 81, 101 and^102^
			Enthaplhy: +50 kJ mol_H_2__^−1^	- Possible cost reduction *vs.* monometallic noble catalysts	- Stability under cycling needs evaluation	
				- Bimetallic nanoparticles exhibit tailored activity enhancements for dehydrogenation, with potential to reduce noble-metal load		
*N*-Heterocycles (*e.g.*, 12H-NEC)	Ru-based alloys	Temperatures of 280–320 °C		- Equivalent Ru activity	- Less widely commercialised relative to Pt/Pd	11, 101 and 103
				- Potential simplification	- Costs are still high	

## 5. Use of catalyst for hydrogenation and dehydrogenation reactions

Various types of catalysts are used for the LOHCs hydrogen storage system. Among these, heterogeneous metal catalysts facilitate hydrogenation and dehydrogenation in LOHC systems under favourable operating conditions.^104^

The hydrogenation of LOHCs is an exothermic reaction, enabling the recovery and integration of excess heat into the overall process design.^19^ Conversely, dehydrogenation is an endothermic process, often catalysed by heterogeneous catalysts at temperatures between 150 and 400 °C and at relatively low pressures, often below 10 bar, contingent upon the individual LOHC system.^25^

Predominantly heterogeneous metal catalysts are used for LOHC hydrogenation to lower activation energy and facilitate hydrogen dissociation and adsorption onto the organic substrate. Noble metals such as Ru, Pd, Pt, and Rh supported on high-surface-area carriers (*e.g.*, Al_2_O_3_, SiO_2_, zeolites) have demonstrated high hydrogenation activity and stability.^105^ These catalysts typically achieve high conversion rates (>80%) under moderate temperatures (150–250 °C) and pressures (30–50 bar), balancing reaction kinetics with energy input.^68^ Ru-based catalysts, in particular, are frequently reported to show superior performance in both hydrogenation and dehydrogenation reactions owing to their effective H_2_ activation, resilience to sintering, and dispersion and electronic properties.^68,106^

Heterogeneous catalysts, including Pt, Pd, Ru, Rh, and Ni, are extensively utilised in hydrogenation and dehydrogenation reactions, with noble metals typically demonstrating superior catalytic activity and selectivity, whilst base metals are frequently explored as economical alternatives.^107^

Benzene and monosubstituted alkyl benzenes have been hydrogenated with a commercial Ni catalyst between 95 and 125 °C and at a pressure of 20–40 atm.^108^ Toluene undergoes hydrogenation at temperatures between 50 and 100 °C and pressures between 10 and 50 bar.^38^ Naphthalene and toluene are hydrogenated using Ni- and PdNi-based catalysts on Si-Al.^109^ An increased resistance to coking was observed during toluene hydrogenation with elevated Ni loadings and the inclusion of Pd in a composite catalyst. Mild hydrogenation conditions have been achieved using a heterogeneous slurry catalytic system comprising metal alloys (LaNi_5_, Mg_2_Ni, and Raney-Ni).^110^

Naphthalene and 1,2,3,4-tetrahydronaphthalene (tetralin) have been hydrogenated in the liquid phase using a Ni catalyst at 80–160 °C and 20–40 bar.^111^ Kirumakki *et al.*^112^ hydrogenated Naphthalene over NiO/SiO_2_–Al_2_O_3_ catalysts with NiO on Al_2_O_3,_ showing the conversion of 90%. Sakanishi *et al.*^113^ found that the catalysts' activity towards the hydrogenation of anthracene and fluorene at a temperature of 100–250 °C and a pressure of 70 bars increased in order of Rh > Pd > Pt > Ru. The carbon and alumina supports behaved similarly during the hydrogenation of polyaromatic hydrocarbons, whereas nano-sized particles enhanced the hydrogenation reactions. Sun *et al.*^114^ reported that nanoRu@hectorite is an efficient and reusable catalyst for the hydrogenation of benzene.

Homogeneous catalytic reactions usually utilise complexes derived from Ru and Ir.^105^ However, rhodium (Rh), iron (Fe), manganese (Mn), and cobalt (Co) complexes have also been investigated, particularly in recent years. Homogeneous catalysts often work at lower temperatures than heterogeneous catalysts.^49^ However, they often require a solvent and may have modest durability. Nonetheless, their utilisation is constrained by high cost, susceptibility to atmospheric conditions, and complexity in recovering the reaction mixture.^105^ In addition, homogeneous hydrogenation of aromatic hydrocarbons is less common than heterogeneous hydrogenation due to lower catalyst efficiency and stability.

The most effective homogenous catalysts for the hydrogenation of aromatic hydrocarbons are complexes containing Ru, Rh or Ir. Several homogeneous Ru catalysts hydrogenate benzene and its derivatives at 90 °C and 60 bar using solvents in biphasic or aqueous systems, with turnover frequencies (TOFs) ranging from 20 to 2000. [Rh(η^5^-C_5_Me_5_)Cl_2_]_2_ can hydrogenate benzene to cyclohexane at 50 °C and 50 bar in the presence of a base, which acts as a promoter and ties up HCl.^115^

Catalytic systems often consist of mixtures of metal salts, including Ni and Co alkoxides, acetylacetonates, or carboxylates, in conjunction with an additional metal component (*e.g.*, Fe, Zn, or Mo), whereas trialkylaluminum is frequently employed as a reducing or activating agent. Aromatic compounds (arenes) can be hydrogenated under catalytic circumstances at temperatures ranging from 155 to 180 °C and hydrogen pressures of approximately 10–30 bar, either in an appropriate solvent or with the substrate in its pure form.^107,116^ For example, 99% conversion of benzene was achieved with a substrate/metal ratio of 18/29.^115^

Tethered complexes on supported metals (TCSM) are combined homogeneous and heterogeneous catalysts. RhCl(PPh_3_)_2_ moieties cross-linked to a phosphonate styrene/divinylbenzene resin have shown activity in the hydrogenation of pyrene, tetralin, p-cresol, and methylnaphthalene. Phenol has been hydrogenated to cyclohexanol with a TOF of 3400 using a Rh(*N*–N)/Pd–SiO_2_ catalyst, whereas both Pd–SiO_2_ and unsupported Rh(*N*–N) are inactive. Hydrogenation has also been conducted using cationic catalysts, such as [Rh(diphos)(cod)](SO_3_CF_3_), on Si-containing supported Pd particles. Surface organometallics are one class capable of arene hydrogenation. Si- or Al-supported Ta, Ti, Zr and Hf hydrides can reduce benzene and alkyl-substituted benzenes with TOFs as high as 1000.^115^

The single-site supported organozirconium catalyst Cp*ZrBz_2_/ZrS (Cp*: Me_5_C_5_, Bz: benzyl, ZrS: sulphated zirconia) catalyses the hydrogenation of arene derivatives to the corresponding cyclohexanes. For example, xylenes were hydrogenated at 25 °C and 7 atm in 1 h.^117^ Some metal-free systems have been shown to activate hydrogen and, consequently, hydrogenate substrates. Frustrated Lewis pairs (FLPs) consisting of Lewis acids and Lewis bases cannot interact directly due to steric hindrance. Still, they can activate molecular hydrogen and deliver the resulting proton (H^+^) and hydride (H^−^) to substrates. Various bases have proved to undergo FLP hydrogenation.^34^ Chernichenko *et al.*^35^ reduced alkynes to alkanes by using FLP under mild conditions at 80 °C and 2 bar using a Ni-based catalyst.^115^

Noble metals efficiently dissociate H_2_ at low activation energies (∼44 kJ mol^−1^), generating reactive hydrogen species for the hydrogenation of aromatic rings.^27,118^ Steric effects influence site selectivity, favouring para-substituted rings over *ortho*-substituted ones. Research employing Density Functional Theory (DFT) indicates a close relationship between catalytic activity and adsorption free energy, where both weak and strong adsorption can hinder catalytic effectiveness.^119,120^ The key rates in hydrogenation involve H_2_ dissociation and C–H bond formation, whereas dehydrogenation emphasises the cleavage of stable aromatic C–H bonds, particularly in DBT.^120^

Despite the effectiveness of noble metals, their scarcity and high cost have motivated research into cheaper transition-metal catalysts such as Ni, Co, Cu, and Mn.^105^ However, their activity and long-term stability still lag those of noble metal systems. Efforts to improve the performance of nonprecious metals include alloying with secondary metals or tailoring the support.

The selection of catalytic support profoundly influences the stability and selectivity of LOHC catalysts.^18,121^ Acidic supports, such as γ-Al_2_O_3_, may induce undesirable side reactions, including coke production, which impairs catalytic efficacy. Conversely, fundamental supports such as MgO or CeO_2_ prolong catalyst longevity by diminishing hydrocarbon polymerisation. Moreover, oxygen vacancies in CeO_2_ facilitate hydrogen spillover to Pd, enhancing catalytic efficiency and longevity. To enhance stability and minimise deactivation during cycles, supplementary modifications are often necessary, such as alkali-metal doping or the development of bifunctional catalyst systems.^121,122^ Consequently, selecting appropriate support material is essential to enhance stability and selectivity in LOHC systems.

Across mature LOHC systems, Pt-group catalysts (Pt, Pd, sometimes Ru) remain the most reported dehydrogenation catalysts, especially for H_18_-DBT. The fixed-bed Pt/Al_2_O_3_ work on H_18_-DBT underscores how catalyst formulation and operating conditions control both hydrogen productivity and by-product formation, highlighting the classic dehydrogenation trade-off- higher driving force can mean higher degradation risk.^89^

However, high activity is insufficient as a selection criterion. A major differentiator is resistance to deactivation under cyclic thermal stress and tolerance to real-world feed impurities. A study on H_18_-DBT shows that gaseous by-products can be dominated by impurities originating from the technical (industrial) LOHC material rather than the idealised molecule itself, directly connecting supply-chain quality to downstream purification burdens.^123^ This finding has two system-level consequences: Dehydrogenation catalyst performance must be assessed with real carrier grades, and LOHC selection cannot ignore the purification, specification, and quality control of the circulating liquid.^123^

### 5.1 Catalyst deactivation and process challenges

A persistent challenge in hydrogenation catalysis is catalyst deactivation, driven by sintering at elevated temperatures, fouling from LOHC impurities, or contamination of the hydrogen feed. Periodic regeneration or replacement of catalysts adds to operating costs and complexity. Additionally, balancing catalyst activity for both hydrogenation and subsequent dehydrogenation is non-trivial- materials highly active for hydrogen uptake may not perform equally well during hydrogen release, necessitating tailored catalysts or dual-bed reactor schemes.^124^

Recent research is exploring bifunctional catalysts that enable efficient hydrogenation and dehydrogenation within a single material, potentially reducing system complexity.^122^ Moreover, advanced supports and promoter elements are being investigated to enhance catalyst dispersion, thermal stability, and resistance to deactivation. Integration of reaction microkinetic modelling and reactor simulation also aids in identifying optimal operating windows and scaling up from laboratory to industrial scales.^19^

The hydrogenation process in LOHC systems relies on carefully engineered catalysts and reactors tailored to achieve high conversion under economically feasible temperatures and pressures. While noble metal catalysts currently lead in performance, cost, and durability, considerations of these factors are driving innovation in catalyst design and reactor integration, which are essential for realising efficient, scalable LOHC hydrogenation.^68^

## 6. Techno-economic and environmental assessment for LOHC

A meaningful evaluation of LOHCs now requires moving beyond technical (*i.e.*, molecular and catalyst) performance and towards system-level competitiveness, where techno-economic analysis (TEA) and lifecycle assessment (LCA) determine whether an LOHC pathway can compete with compressed H_2_, liquid H_2_, or ammonia under realistic logistics. LOHC feasibility is rarely constrained by hydrogenation chemistry alone; instead, it is dominated by (i) the energy and hardware needed to release H_2_ (dehydrogenation/heat supply), (ii) carrier makeup and losses, (iii) hydrogen purification requirements, and (iv) the carbon intensity and cost of electricity/heat used across the chain.

A comparative cost analysis shows that LOHC routes are very competitive with NH_3_ in many scenarios, and a cost-uncertainty analysis indicates that material-related costs (including LOHC fluids) can significantly influence levelised costs.^125^ Economic penalties, including dehydrogenation heat duty, catalyst lifetime, and downstream purification, as well as the carrier and system used, highly influence the cost of LOHC.^126^ Hydrogen release costs for the TOL-MCH system are generally reported to be $2.08–6.97 kg^−1^ H_2_.^127,128^ Conversely, the NEC system was reported to have significantly lower hydrogen release costs ranging from $0.09 to 0.9 kg_H_2__^−1^,^129^ mainly due to lower dehydrogenation temperatures and the corresponding lower operating expenses. However, the values are highly dependent on the system and scenario assumptions used in the TEA studies. The DBT systems also reported a cost of $4.21 kg_H_2__^−1^,^130^ where the higher heat demand associated with the dehydrogenation and hydrogen purification process contributes significantly to the overall operating expenses.

It can also be noted that the cost values reported for the different systems are not necessarily dependent on the specific molecules used in the LOHC systems, as the values are also a function of the thermodynamic enthalpy associated with the dehydrogenation process, the temperature control, the catalyst durability, and the carrier degradation rates considered in the TEA studies. Thus, the TEA studies are not necessarily a comparison of the carrier used but rather a comparison of the heat management strategy, the reactor design, and the overall operating durability considered in the TEA studies.

Several system studies indicate that when the logistics context strongly favours liquid-fuel-like handling (existing tanks, pipelines, and shipping interfaces) and when waste heat or low-cost heat is available, LOHCs can improve their position relative to cryogenic H_2_, or high-pressure distribution. This is why industrial export/import chains and large-scale storage are frequently highlighted as LOHC best-fit domains rather than universal solutions.^131^ Not all TEA studies consistently address carrier degradation, makeup rates, and hydrogen purification, yet experimental evidence shows that technical-grade LOHC fluids can introduce impurities that increase purification burden and, therefore, system cost and energy use.^27^

Toxicity, flash point, and biodegradability are increasingly prominent in screening frameworks, especially for large-scale or mobile applications where human exposure and ecological impacts matter. LCA studies increasingly consider LOHCs as transport enablers for renewable hydrogen, whereas environmental sustainability depends strongly on how the carrier is produced and the energy sources used. A recent LCA of renewable hydrogen transport using LOHCs explicitly evaluates renewable toluene and renewable dibenzyltoluene (produced from biomass feedstocks) as carriers in long-distance shipping scenarios, emphasising that upstream carrier production pathways and transport distance materially affect the environmental profile.^27^ Other LCA work directly compares TOL-MCH Vs DBT-PHDBT (H18-DBT) chains in European logistics contexts, underscoring that carrier choice shifts the environmental hotspots. DBT pathways may gain from handling properties but can be penalised through energy demand and supply-chain assumptions.^132^

LOHCs can look attractive in TEA if logistics are smooth, but can still perform poorly in LCA if (a) dehydrogenation heat is fossil-based, (b) carrier production is carbon-intensive, or (c) purification/recirculation losses are high. Conversely, green LOHC (bio-derived or low-carbon-synthesised carriers) can meaningfully improve LCA outcomes but may introduce new cost and supply constraints.^133^ Most related studies emphasise the need to reduce both costs and emissions, focusing on heat management, intensified dehydrogenation, catalyst durability, and more innovative system design.^131^

As dehydrogenation is endothermic, strategies that reduce external heat input *via* waste heat integration, intensified heating methods, or improved heat-transfer architectures are repeatedly recognised as essential for scale-up.^10^ In addition, microstructure and intensified reactor concepts are being highlighted to address the heat bottleneck and improve hydrogen release productivity, thereby directly targeting the dominant OPEX/CAPEX drivers in many TEAs and also lowering emissions by more efficiently using heat.^10^

From a life-cycle perspective, the environmental performance of hydrogen storage technologies is influenced by hydrogen production and the energy required for storage, transportation, and release. For LOHC systems, hydrogenation requires 2–6 kWh kg_H_2__^−1^ and dehydrogenation requires 8–15 kWh kg_H_2__^−1^, leading to an overall energy penalty of 10–20 kWh kg_H_2__^−1^, which can be partially reduced by heat recovery.^17,43^ By comparison, compressed hydrogen storage consumes 2–4 kWh kg_H_2__^−1^ to compress to 700 bar, with a carbon footprint of 80–160 g CO_2_e kg_H_2__^−1^ (assuming a low emission factor of ∼40 g CO_2e_/kW h^−1^). Liquid hydrogen has a larger footprint of 400–500 g CO_2e_ per kg H_2_ due to cryogenic processes, which require 10–13 kWh kg_H_2__^−1^.^40^

Ammonia is a widely used hydrogen carrier. However, catalytic ammonia cracking is highly endothermic and typically incurs an overall energy penalty of approximately 15–20%. When fossil-derived heat is used, the cracking stage can increase LCA emissions by approximately 1–3 kg CO_2_e kg_H_2__^−1^, whereas renewable electricity or waste heat can substantially reduce these additional emissions.^134,135^

Moreover, methanol synthesis requires capture, and its round-trip efficiency rarely exceeds 50%, thereby elevating its total life-cycle footprint unless strictly renewable energy and green technologies are employed. Greenhouse gas emissions vary depending on the hydrogen production routes and the carbon intensity of the energy used for hydrogenation and dehydrogenation. Therefore, the choice of storage technology must be based on a thorough evaluation of storage capacity, efficiency, energy consumption, emissions, logistics and compatibility with infrastructure.

Moreover, machine learning (ML) is increasingly being applied to hydrogen technologies; however, existing reviews indicate that most ML studies remain confined to specific subdomains, while theory-driven approaches and deployment-oriented datasets remain underdeveloped.^136^ For LOHC-relevant catalysis specifically, physics-based ML has been applied to design bimetallic catalysts for dehydrogenating hydrogen carriers, with the explicit goal of improving rates while reducing precious-metal usage.^137^Table 6 compares TEA and LCA for two different LOHC systems. Based on this table, both the Toluene-MCH and DBT-H_18_-DBT LOHC processes demonstrate significant sensitivity to techno-economic and environmental performance factors, including heat availability, carrier delivery route, and system boundary assumptions, and the energy requirement for hydrogen production from the dehydrogenation step becomes an important contributor to costs and emissions.

**Table 6:** Comparison of TEA and LCA concerning Toluene-MCH and DBT-H_18_-DBT LOHCs systems

Parameters	Toluene–MCH	DBT-H18-DBT	Summary of TEA/LCA	Advancement
System	Export/transport chains and supply logistics (mature benchmark LOHC)^132^	Large-scale storage/transport; attractive handling and stability; widely studied in practice reviews^131^	Full-chain costing and emissions depend on heat/electricity sources and logistics assumptions^126^	Integrated supply-chain design and deployment-focused demonstrations^138^
Economics	Dehydrogenation heat and purification dominate overall penalties^10^	Carrier/handling and purity assumptions become decisive in cost realism^27^	Competing carriers (notably ammonia) often show a cost advantage in comparative analyses.^125^ Cost: Toluene-methylcyclohexane (MCH) system: $2.083 – $6.97/kg H_2_ (ref. 127 and 128)	Thermal neutrality/heat intensification; catalyst lifetime improvement.^93^
			*N*-Ethylcarbazole (NEC) system: $0.09–$0.9/kg H_2_.^129^ Dibenzyltoluene (DBT) system: 4.21/kg H_2_.^130^	
LCA	Upstream energy + logistics + carrier pathway (mainly if fossil heat is used)^132^	Carrier pathway and dehydrogenation energy; outcomes are sensitive to “green LOHC” assumptions^133^	Carrier production route (bio-derived *vs.* conventional) can swing results markedly^133^	Low-carbon heat/electricity coupling; green carrier synthesis routes^133^
Future direction	Better dehydrogenation heat supply and intensified reactors^93^	Heat-intensified dehydrogenation + impurity-tolerant, durable catalysts^93^	Innovations must reduce both energy penalty and cost volatility	Physics-inspired ML catalyst design + deployment-grade datasets; thermal-neutral architectures^139^

## 7. Advancement, challenges and future direction for LOHCs

LOHCs represent a promising chemical hydrogen storage pathway due to their ability to reversibly bind and release hydrogen under comparatively mild conditions while remaining liquid at ambient temperatures, thereby simplifying handling, transport, and integration with existing infrastructure.^11^ The reversible pair concept of hydrogenation and dehydrogenation has enabled extensive research into specific carrier molecules and their associated challenges.^27^

Recently, electrocatalytic methods for LOHCs have been studied, in which hydrogenation and dehydrogenation are performed using primarily electricity and electrocatalysts.^25^ The electrocatalytic method entails hydrogenating the hydrogen-deficient LOHC at the cathode and facilitating oxidising reactions at the anode, where the hydrogen-saturated molecule undergoes dehydrogenation, releasing hydrogen from the cathode.^140^ This approach provides benefits, including the *in situ* generation of hydrogen at the cathode surface, enabling reactions under mild conditions of ambient temperature and atmospheric pressure, and circumventing diffusion constraints associated with insoluble hydrogen.^141,142^ Nonetheless, a notable disadvantage is the conflict between hydrogenation and hydrogen evolution, where a low ratio of hydrogenation to hydrogen desorption rates can lead to inefficient processes.^142^ Efficiency enhancements can be achieved through the use of ionic liquids as electrolytes and the optimisation of membrane materials; research has demonstrated superior performance with sulfonated poly(arylene ether sulfone) membranes compared to conventional Nafion membranes.^143,144^ Additionally, a dual approach formulated by Sievi *et al.*^145^ amalgamates thermocatalytic and electrocatalytic techniques to facilitate hydrogen utilisation without external thermal energy, utilising transfer hydrogenation and fuel cell technology. Electrocatalytic hydrogen storage capabilities encompass a range of organic molecules, highlighting progress in hydrogen utilisation technology.^146^

Recently, dibenzyl toluenes (DBT a6) and benzyl toluenes (BT a7), common heat-transfer fluids known under the commercial names Marlotherm® SH (a6) and Marlotherm® LH (a7), were proposed as LOHCs.^14,38^ The hydrogen storage capacity of DBT is 6.2 wt%, equivalent to 57 kg_H_2__ m^−3^ (624 N m^3^).^19^ The working temperature of DBT is 70–380 °C.^14^ Hydrogenous GmbH has commercialised DBT as LOHC. Hynertech, a prominent Chinese company specialising in LOHC technology, has established a 1000-ton *N*-ethyl carbazole (NEC) facility and unveiled the world's first fuel cell model powered by liquid organic hydrogen.^19,147^ In 2020, Germany initiated the “GET H2” project to develop large-scale green hydrogen production in areas rich in wind and solar energy. The project aims to incorporate downstream LOHC technology, marking a substantial advancement in energy storage within renewable systems.^19,148^

Aromatic systems often struggle with high dehydrogenation temperatures, while heterocyclic carriers may impose complex catalyst requirements and synthesis challenges.^60^ Future research should focus on advancing rational molecular design through thermodynamic modelling, high-throughput screening, and integrated assessment frameworks that combine molecular performance with techno-economic and life cycle sustainability metrics. This holistic perspective is essential to advancing LOHCs from promising laboratory systems to robust components of a sustainable hydrogen infrastructure. Additionally, economic and supply factors, including feedstock availability, synthesis costs, and catalyst requirements, shape practical selection, highlighting dibenzyltoluene for its high hydrogen storage density and industrial readiness.^149^

Among extensively studied LOHC couples, the toluene-methylcyclohexane (TOL-MCH) system stands out as one of the earliest developed and best characterised. In this approach, toluene (LOHC^−^) is hydrogenated to methylcyclohexane (LOHC^+^), which can be stored or transported and later dehydrogenated to liberate hydrogen. The TOL-MCH system benefits from established catalytic pathways and relatively high volumetric hydrogen density but suffers from high dehydrogenation temperatures (typically exceeding 300 °C) and significant endothermic heat requirements for hydrogen release. These energy penalties reduce overall cycle efficiency and necessitate robust heat management strategies in practical reactors.^27^

**Fig. 4:**
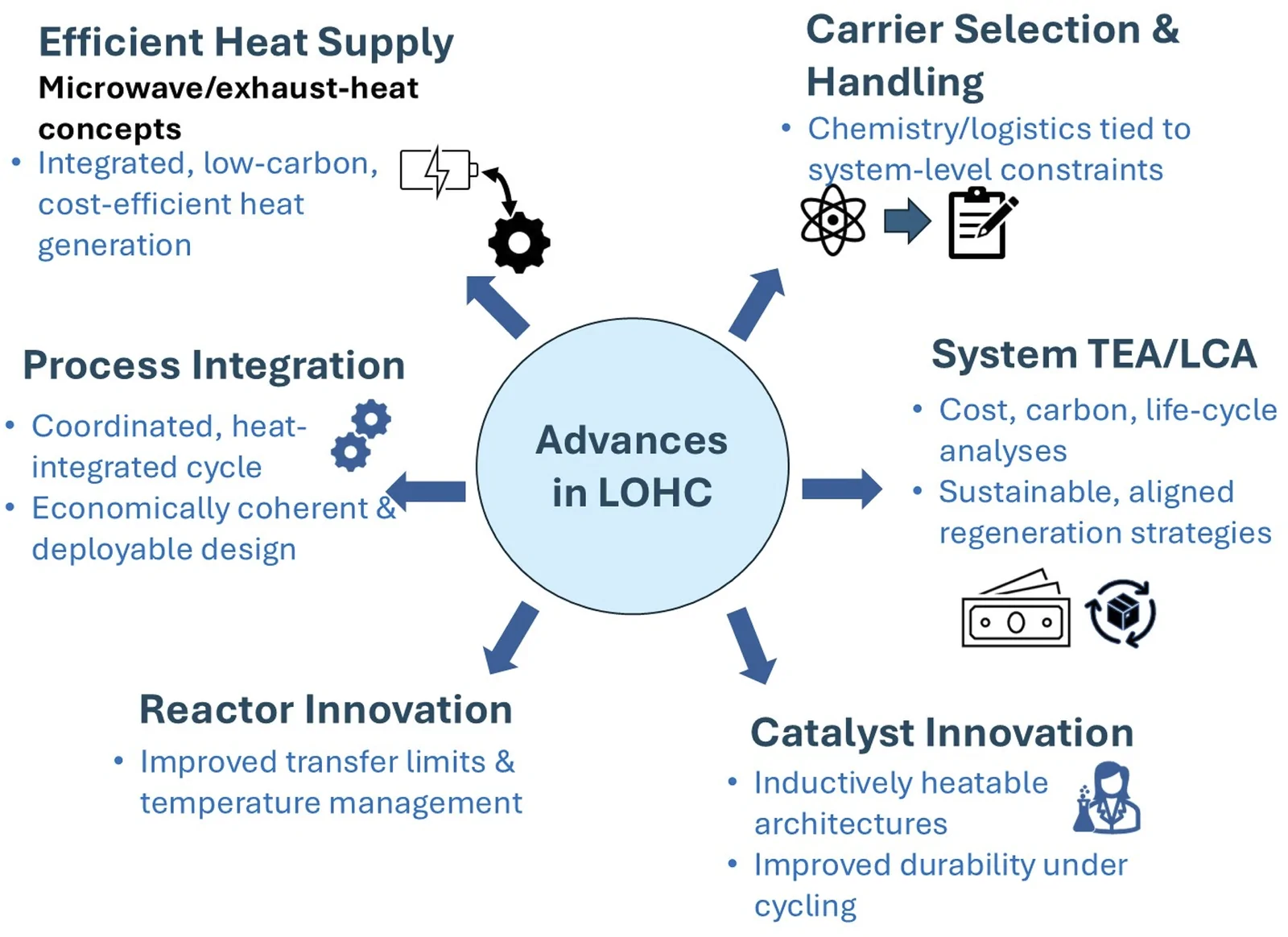
Advancements in LOHC.

The multidimensional nature of LOHC advancement is schematically depicted in Fig. 4, with special emphasis on the transition from chemistry validation to system-level engineering to achieve net efficiency, durability, and deploy ability. In particular, the figure emphasises the interdependencies among thermal management, reactor intensification, catalyst durability, and carrier strategy.

Beyond these widely used standard systems, emerging LOHC molecules such as cyclic naphthalene/decalin pairs and heteroaromatic structures have been proposed to tailor hydrogen capacity, reaction thermodynamics, and fluid properties. Such novel carriers aim to balance hydrogen capacity, dehydrogenation energetics, and liquid-handling characteristics, but often require specialised catalysts or face scalability issues during synthesis.^150^

A key trend in LOHC research is the integration of carrier design with catalyst and reactor engineering. The catalytic conversion steps govern both hydrogenation efficiency and dehydrogenation selectivity. Thus, many studies now focus on metal catalysts (*e.g.*, Pd, Ru, Ni) supported on tailored substrates to enhance activity, reduce activation energy, and suppress side reactions such as cracking or coke formation. Optimising catalyst morphology, dispersion, and support interactions has shown promise in lowering operational temperatures and improving long-term stability.^150^

Despite these advances, significant challenges remain. The high energy demand of dehydrogenation, catalyst deactivation over repeated cycles, carrier degradation, and the balance between hydrogen capacity and fluid properties are major research bottlenecks. Moreover, the holistic assessment of LOHC performance, including catalyst costs, reactor heat integration, and lifecycle environmental impacts, is essential to validate LOHC technology against competing storage solutions such as compressed hydrogen, metal hydrides, and liquid carriers (*e.g.*, ammonia).^126^

To overcome some limitations of simple aromatics, research has intensively focused on larger, more complex carriers such as DBT and its perhydro derivative (18H-DBT). DBT-based systems are widely recognised for their enhanced thermal stability, low volatility, and a wide liquid-phase range*,* which are crucial for long-distance transport and industrial logistics. Furthermore, DBT systems have competitive hydrogen storage densities (∼6 wt%), and their physical properties (low vapour pressure, high flash point) offer critical safety advantages relative to lighter aromatics. Nonetheless, efficient dehydrogenation still requires temperatures typically over 250 °C, and catalyst deactivation due to thermal cycling remains a significant operational issue that must be addressed for long-term deployment.^26^

While systems such as TOL-MCH, DBT-based carriers, and NEC continue to dominate state-of-the-art research, the field's trajectory points toward molecular innovation, accompanied by advanced catalytic and reactor solutions to address remaining efficiency and scalability challenges. The interplay of molecular chemistry, catalytic engineering, and systems integration will likely define the next generation of LOHC technologies, shaping their role in a future sustainable hydrogen economy.^27^ Moreover, how efficiently and economically H_2_ can be released at the point of use while maintaining catalyst life and hydrogen quality across many cycles is a vital question.^27^ Application-focused research on thermal neutrality encompasses enhanced heating methods (such as microwave heating), exhaust heat recirculation, and system designs that mitigate substantial external heat requirements.^93^ Moreover, dehydrogenation is a Multiphysics problem; for example, the reaction produces large volumes of gas within a liquid, challenging wetting, heat transfer, and catalyst contact. Intensified reactors (*e.g.*, microchannels/continuous concepts) can improve transfer limits and temperature management, which is why reactor innovation is now equally important with catalyst development.^11^

Some nitrogen-containing LOHCs, while offering attractive hydrogen capacities, must be evaluated for such properties to ensure commercial viability.^60^

For NEC-type systems, for example, process integration studies discuss linking hydrogenation and dehydrogenation in a coordinated way, reinforcing that system design can matter as much as catalyst selection.^151^

Critically, this is not only a materials issue; it becomes a supply-chain and operations issue (carrier specification, impurity management, regeneration strategy), and this is often underestimated. Recent advancements have demonstrated inductively heatable catalyst architectures for the dehydrogenation of perhydrodibenzyltoluene, which are conceptually significant because they provide heat in proximity to active sites, potentially diminishing thermal inertia and enhancing controllability relative to solely externally heated beds.^91^

A recent study stresses that carrier selection must be tied to system-level constraints (available heat grade, target purity, logistics).^11^ For example, hydrocarbon carriers (*e.g.*, MCH, DBT family) can be attractive for logistics and handling but are often heat-demanding for dehydrogenation.^93^ N-heterocycles (*e.g.*, NEC family) are widely studied, partly because they can enable milder conditions, but they can introduce chemistry- and catalyst-sensitivity issues (selectivity, by-products, stepwise hydrogenation complexity).^152^ Techno-economics and LCA must be coupled to engineering reality. Various studies explicitly integrate TEA/LCA considerations into discussions of catalysts, reactors, and supply chains, rather than treating sustainability and cost as afterthoughts.^11^ Many TEA/LCA studies still require greater alignment with validated durability metrics, realistic regeneration frequencies, and realistic heat-integration architectures. Table 7 outlines a roadmap to tackle challenges associated with the LOHC.

**Table 7:** Roadmap to tackle challenges associated with the LOHC

Theme	Advancement	Limitations	Roadmap	Applications	Ref.
Thermal neutrality/heat supply	Microwave/exhaust-heat concepts; electrified/local heating; induction approaches	Endothermic dehydrogenation energy penalty; heat transfer limits	Demonstrate net-efficient heat integration and electrified heating with full energy accounting	Industrial hubs with waste heat; electrified stations	93
Reactor/process intensification	Continuous/intensified reactors; improved transfer control	Gas evolution and liquid wetting reduce effective contact; scale-up complexity	Scale continuous reactors with validated control & durability	High-throughput release units; modular deployments	126
Catalyst innovation	Tailored catalyst architectures: Durability focus	Deactivation over cycling; sensitivity to real feeds/carrier impurities	Shift metrics from activity to lifetime and regenerability	Any LOHC deployment	27
Carrier molecule strategy	Broader material portfolios; “lower enthalpy carriers” concept	No universal best carrier; trade-offs between heat demand and chemical sensitivity	Match carriers to heat-grade availability and purity needs; standardise specs	DBT-like for logistics; NEC-like, where a lower temperature is crucial	105
System TEA/LCA	More lifecycle/chain-level reviews	TEA/LCA often lacks durability/operations realism	Bankable demos: validated cycles, supply chain specs and safety standards	Early industrial corridors; demonstration refuelling corridors	11

## 8. Conclusions

Liquid organic hydrogen carriers (LOHCs) enable reversible storage and transport of hydrogen at ambient conditions using existing liquid-fuel infrastructure. The most mature LOHC candidates, including hydrocarbon systems such as toluene/MCH and high-boiling carriers such as perhydro-DBT, as well as N-heterocyclic carriers such as *N*-ethylcarbazole, demonstrate that reversible hydrogenation/dehydrogenation is technically feasible and scalable. However, the molecular design must account for trade-offs among storage capacity, dehydrogenation temperature, reaction thermodynamics, stability, viscosity, safety, and cost.

The storage capacity of commercial LOHCs, such as toluene/methylcyclohexane and dibenzyl toluene, is about 6.2 wt% H_2_ and the best reversible systems, naphthalene/decalin and biphenyl/bicyclohexyl, achieve ∼7.3 wt%. Hydrogenation requires a temperature of 180–225 °C for toluene/MCH and NEC systems, while a much higher temperature of ∼300 °C for BT and DBT systems. However, dehydrogenation is a much more energy-intensive process, typically occurring at 250–450 °C for MCH systems and 280–320 °C for BT, DBT, and NEC systems.

A practical theoretical limit is estimated for stable reversible organic liquids at ∼9 wt% H_2_, but it is still difficult to achieve simultaneously high hydrogen capacity, low dehydrogenation temperature (<170–180 °C), low viscosity (<5 mPa s), wide liquid operating range (−20 to >250 °C), long cycle life (>10 000 cycles) and low cost. Ammonia has a significantly higher hydrogen content (17.6 wt%) and volumetric hydrogen density (∼108 kg_H_2__ m^−3^) than LOHCs (55–65 kg_H_2__ m^−3^), but its toxicity, high cracking temperature (500–700 °C), and hydrogen purification requirements limit its widespread use compared to LOHCs.

This review highlights that the key challenge for commercial deployment is not hydrogen loading or hydrogenation but efficient hydrogen releases due to its endothermic heat requirement, transport limitations in multiphase reactors, and catalyst durability issues under cyclic operation. Hydrogenation/dehydrogenation processes usually involve noble-metal catalysts such as Pt, Pd, and Ru supported on oxide-based materials. Various catalyst formulations, including Pt/KIT-6, Pt/TiO_2_, Pt/Al_2_O_3_, Ru/Al_2_O_3_, Pt/V_2_O_5_, Pt/Y_2_O_3_, and Pt–Re/Al_2_O_3_, have been examined; however, coking, sintering, and catalyst deactivation remain major issues.

No single LOHC is universally optimal across hydrogen storage capacity, reaction thermodynamics, dehydrogenation kinetics, physicochemical properties, stability, safety, cost, and overall system performance. H–C carriers offer strong infrastructure compatibility and handling advantages that typically require higher dehydrogenation temperatures, while heterocyclic carriers enable low temperature release but suffer from catalyst design, impurities, and by-product formation. Thus, LOHC selection must be application-driven, considering heat availability, hydrogen purity requirements, scale, and supply chain constraints.

From a techno-economic perspective, LOHC technology still requires significant capital and energy inputs, as reflected in estimated levelized costs of $2.0–7.0/kg H_2_ for the toluene/MCH system and $4.0/kg H_2_ for the DBT system.

With continued progress in catalyst stability, reactor heat integration, and infrastructure alignment, LOHCs can become a key enabling technology for reliable hydrogen storage and distribution in the evolving low-carbon energy economy. Ongoing research should therefore focus on system-level innovation, including improved catalyst architectures, intensified reactor concepts, and thermal-neutrality strategies that recover or deliver heat more effectively to the reaction zone. Furthermore, future research should focus on AI-guided inverse molecular design, high-throughput DFT screening, heteroatom-engineered molecular frameworks, earth-abundant low-cost catalysts, and integrated molecule-catalyst co-design to discover next-generation LOHCs with improved hydrogen capacity, lower energy requirements, enhanced durability, and superior techno-economic performance.

## Consent to publish declaration

We hereby declare that all authors have read and approved the final version of this manuscript and consent to its submission and publication. We confirm that the work is original and has not been published or submitted elsewhere. All authors have given their consent to publish this work.

## Conflicts of interest

The authors declare that they have no known competing financial interests or personal relationships that could have influenced the work reported in this paper.

## Data Availability

No primary research results, software or code have been included and no new data were generated or analysed as part of this review.

## References

[cit1] Anekwe I. M. S. (2025). *et al.*, The hydrogen challenge: addressing storage, safety, and environmental concerns in hydrogen economy. Int. J. Hydrogen Energy.

[cit2] Shahabuddin M. (2020). *et al.*, Advances in the thermo-chemical production of hydrogen from biomass and residual wastes: Summary of recent techno-economic analyses. Bioresour. Technol..

[cit3] Aakko-Saksa P. T. (2018). *et al.*, Liquid organic hydrogen carriers for transportation and storing of renewable energy – Review and discussion. J. Power Sources.

[cit4] Geburtig D. (2016). *et al.*, Chemical utilization of hydrogen from fluctuating energy sources–Catalytic transfer hydrogenation from charged Liquid Organic Hydrogen Carrier systems. Int. J. Hydrogen Energy.

[cit5] Markiewicz M. (2015). *et al.*, Environmental and health impact assessment of Liquid Organic Hydrogen Carrier (LOHC) systems–challenges and preliminary results. Energy Environ. Sci..

[cit6] Makepeace J. W. (2019). *et al.*, Reversible ammonia-based and liquid organic hydrogen carriers for high-density hydrogen storage: Recent progress. Int. J. Hydrogen Energy.

[cit7] Rao N., Lele A. K., Patwardhan A. W. (2022). Optimization of liquid organic hydrogen carrier (LOHC) dehydrogenation system. Int. J. Hydrogen Energy.

[cit8] Reymond A. (2025). *et al.,* Liquid Organic Hydrogen Carriers (LOHCs): Comprehensive Comparison and Future Outlook. Energy Fuels.

[cit9] Abedin Khan Z. (2024). *et al.*, A review on sustainable use of recycled construction and demolition waste aggregates in pavement base and subbase layers. Cleaner Mater..

[cit10] Chu C. (2023). *et al.*, Hydrogen storage by liquid organic hydrogen carriers: Catalyst, renewable carrier, and technology – A review. Carbon Resour. Convers..

[cit11] Lin A., Bagnato G. (2024). Revolutionising energy storage: The Latest Breakthrough in liquid organic hydrogen carriers. Int. J. Hydrogen Energy.

[cit12] Ramadhani S. (2024). *et al.*, Advances in catalytic hydrogenation of liquid organic hydrogen carriers (LOHCs) using high-purity and low-purity hydrogen. ChemCatChem.

[cit13] Agyekum E. B., Tarawneh B., Rashid F. L. (2025). State-of-the-Art review of liquid organic hydrogen carriers for hydrogen storage and transport: challenges and perspectives. Appl. Energy.

[cit14] Müller K. (2015). *et al.*, Liquid organic hydrogen carriers: thermophysical and thermochemical studies of benzyl-and dibenzyl-toluene derivatives. Ind. Eng. Chem. Res..

[cit15] Pei P. (2025). *et al.*, Enhanced ambient-temperature hydrogen physisorption in porous graphene materials with defect-stabilized atomic potassium. Int. J. Hydrogen Energy.

[cit16] Alharthi L. (2026). *et al.*, Hydrogen storage in carbon materials: A review of mechanisms, performance, and optimization strategies. Renewable Sustainable Energy Rev..

[cit17] Fikrt A. (2017). *et al.*, Dynamic power supply by hydrogen bound to a liquid organic hydrogen carrier. Appl. Energy.

[cit18] Lee J. (2024). *et al.*, Development of dehydrogenation system for liquid organic hydrogen carrier with enhanced reaction rate. Appl. Sci..

[cit19] Rakić E., Grilc M., Likozar B. (2023). Liquid organic hydrogen carrier hydrogenation–dehydrogenation: From ab initio catalysis to reaction micro-kinetics modelling. Chem. Eng. J..

[cit20] Bong B. (2025). *et al.*, Reaction Equilibria in the Hydrogen Loading and Release of the LOHC System Benzyltoluene/Perhydro Benzyltoluene. Chem. Eng. Technol..

[cit21] Lederhos C. R. (2013). *et al.*, Metal and precursor effect during 1-heptyne selective hydrogenation using an activated carbon as support. Sci. World J..

[cit22] Ma C. (2025). *et al.*, Tuning the oxidative dehydrogenation of propane mechanism by Pd–B/Al2O3bifunctional catalysis through suppression of gas-phase radicals and enhancement of surface-mediated pathways. Catal.:Sci. Technol..

[cit23] Caravati M., Grunwaldt J. D., Baiker A. (2007). Comparative in situ XAS investigations during aerobic oxidation of alcohols over ruthenium, platinum and palladium catalysts in supercritical CO2. Catal. Today.

[cit24] Liu Y. (2014). *et al.*, Reversible hydrogen storage behavior of LiBH4-Mg(OH)2 composites. Int. J.
Hydrogen Energy.

[cit25] Barthos R. (2025). *et al.*, Catalytic Aspects of Liquid Organic Hydrogen Carrier Technology. Catalysts.

[cit26] Jangir J., Jagirdar B. R. (2025). Unveiling the Potential of Heterogeneous Systems for Reversible Hydrogen Storage in Liquid Organic Hydrogen Carriers. ChemSusChem.

[cit27] Daud M. U. (2025). *et al.*, Liquid organic hydrogen carriers (LOHC): from definitions to recent developments. Rend. Lincei Sci. Fis. Nat..

[cit28] Jeong K. (2024). *et al.*, Benzyl-methylbenzyl-benzene: Improving hydrogen storage and release performance of dibenzyltoluene based liquid organic hydrogen carrier. Chem. Eng. J..

[cit29] Osman A. I. (2024). *et al.*, Advances in hydrogen storage materials: harnessing innovative technology, from machine learning to computational chemistry, for energy storage solutions. Int. J. Hydrogen Energy.

[cit30] Lavanya M. (2024). *et al.*, An overview of hydrogen storage technologies–Key challenges and opportunities. Mater. Chem. Phys..

[cit31] Otsuka K. (2003). *et al.*, Chemical storage of hydrogen by modified iron oxides. J. Power Sources.

[cit32] Züttel A. (2004). Hydrogen storage methods. Naturwissenschaften.

[cit33] Hassan I. A. (2021). *et al.*, Hydrogen storage technologies for stationary and mobile applications: Review, analysis and perspectives. Renewable Sustainable Energy Rev..

[cit34] Hu Y. H., Zhang L. (2010). Hydrogen storage in metal–organic frameworks. Adv. Mater..

[cit35] Zhao Y. (2026). *et al.*, Constrained carbon bonding inside fullerene cages. Chem. Soc. Rev..

[cit36] Wu Q. (2020). *et al.*, Carbon-based nanocages: a new platform for advanced energy storage and conversion. Adv. Mater..

[cit37] Reuß M. (2017). *et al.*, Seasonal storage and alternative carriers: A flexible hydrogen supply chain model. Appl. Energy.

[cit38] Schneider M. J. Hydrogen storage and distribution via liquid organic carriers. in Bridging Renewable Electricity with Transportation Fuels Workshop, Brown Palace Hotel, Denver, CO, 2015

[cit39] Mekonnin A. S. (2025). *et al.*, Hydrogen Storage Technology, and Its Challenges: A Review. Catalysts.

[cit40] IEA , Global Hydrogen Review. International Energy Agency, 2025, Available at: https://www.iea.org/reports/global-hydrogen-review-2025

[cit41] Maigret De , Macchi E. G. J., and Blanco H., Global hydrogen trade to meet the 1.5 C climate goal: Part III–green hydrogen supply cost and potential. in Global Hydrogen Trade to Meet the 1.5 C Climate Goal. 2022

[cit42] Abdin Z., Khalilpour K., Catchpole K. (2022). Projecting the levelized cost of large scale hydrogen storage for stationary applications. Energy Convers. Manage..

[cit43] Mehr A. S. (2024). *et al.*, Recent challenges and development of technical and technoeconomic aspects for hydrogen storage, insights at different scales; A state of art review. Int. J. Hydrogen Energy.

[cit44] Xu Z. (2022). *et al.*, Techno-economic analysis of hydrogen storage technologies for railway engineering: a review. Energies.

[cit45] Boretti A. (2024). Technology readiness level of hydrogen storage technologies for transport. Energy Storage.

[cit46] Preuster P., Papp C., Wasserscheid P. (2017). Liquid organic hydrogen carriers (LOHCs): toward a hydrogen-free hydrogen economy. Acc. Chem. Res..

[cit47] Niermann M. (2019). *et al.*, Liquid Organic Hydrogen Carrier (LOHC) – Assessment based on chemical and economic properties. Int. J. Hydrogen Energy.

[cit48] Moores A. (2006). *et al.*, Catalysed low temperature H 2 release from nitrogen heterocycles. New J. Chem..

[cit49] Crabtree R. H. (2008). Hydrogen storage in liquid organic heterocycles. Energy Environ. Sci..

[cit50] Emel’yanenko V. N. (2015). *et al.*, Hydrogen storage: thermochemical studies of N-alkylcarbazoles and their derivatives as a potential liquid organic hydrogen carriers. J. Phys. Chem. C.

[cit51] Stark K. (2015). *et al.*, Liquid organic hydrogen carriers: thermophysical and thermochemical studies of carbazole partly and fully hydrogenated derivatives. Ind. Eng. Chem. Res..

[cit52] Heller A. (2016). *et al.*, Binary diffusion coefficients of the liquid organic hydrogen carrier system dibenzyltoluene/perhydrodibenzyltoluene. J. Chem. Eng. Data.

[cit53] Aslam R. (2016). *et al.*, Measurement of hydrogen solubility in potential Liquid Organic Hydrogen Carriers. J. Chem. Eng. Data.

[cit54] Aslam R., Müller K., Arlt W. (2015). Experimental study of solubility of water in Liquid Organic Hydrogen Carriers. J. Chem. Eng. Data.

[cit55] Stark K. (2016). *et al.*, Melting points of potential liquid organic hydrogen carrier systems consisting of N-alkylcarbazoles. J. Chem. Eng. Data.

[cit56] Wang H., Zhou X., Ouyang M. (2016). Efficiency analysis of novel Liquid Organic Hydrogen Carrier technology and comparison with high pressure storage pathway. Int. J. Hydrogen Energy.

[cit57] Müller K., Geng J., Arlt W. (2013). Reversible vs. irreversible conversion of hydrogen: how to store energy efficiently?. Energy Technol..

[cit58] Adametz P. (2017). *et al.*, Thermodynamic Evaluation and Carbon Footprint Analysis of the Application of Hydrogen-Based Energy-Storage Systems in Residential Buildings. Energy Technol..

[cit59] Pradhan A. U. (2011). *et al.*, A feasibility analysis of hydrogen delivery system using liquid organic hydrides. Int. J. Hydrogen Energy.

[cit60] Rao P. C., Yoon M. (2020). Potential Liquid-Organic Hydrogen Carrier (LOHC) Systems: A Review on Recent Progress. Energies.

[cit61] Ndhlozi L. (2025). *et al.*, Cyclic liquid organic hydrogen carriers for efficient hydrogen storage using mesoporous catalytic systems. Discover Energy.

[cit62] Do G. (2016). *et al.*, Hydrogenation of the liquid organic hydrogen carrier compound dibenzyltoluene–reaction pathway determination by 1 H NMR spectroscopy. React. Chem. Eng..

[cit63] Aslam R. (2016). *et al.*, Development of a liquid chromatographic method for the separation of a liquid organic hydrogen carrier mixture. Sep. Purif. Technol..

[cit64] Müller K. (2016). *et al.*, Experimental assessment of the degree of hydrogen loading for the dibenzyl toluene based LOHC system. Int. J. Hydrogen Energy.

[cit65] Inhetveen P., Alt N., Schluecker E. (2016). Measurement of the hydrogenation level of dibenzyltoluene in an innovative energy storage system. Vib. Spectrosc..

[cit66] Modisha P. M. (2019). *et al.*, The prospect of hydrogen storage using liquid organic hydrogen carriers. Energy Fuels.

[cit67] Garidzirai R., Modisha P., Bessarabov D. (2023). Assessment of reaction kinetics for the dehydrogenation of perhydro-dibenzyltoluene using Mg-and Zn-modified Pt/Al2O3 catalysts. Catalysts.

[cit68] Permude P. (2025). *et al.*, Effective catalysts for typical liquid organic hydrogen carrier N-Ethylcarbazole. Int. J. Hydrogen Energy.

[cit69] Bong B. (2025). *et al.*, Reaction Equilibria in the Hydrogen Loading and Release of the LOHC System Benzyltoluene/Perhydro Benzyltoluene. Chem. Eng. Technol..

[cit70] Yang Y. (2022). *et al.*, Study on high hydrogen yield for large-scale hydrogen fuel storage and transportation based on liquid organic hydrogen carrier reactor. Fuel.

[cit71] Yadav V. (2021). *et al.*, Recent advances in liquid organic hydrogen carriers: an alcohol-based hydrogen economy. ACS Catal..

[cit72] Lippert J., Wasserscheid P., Geißelbrecht M. (2026). Continuous hydrogenation of the hydrogen storage compound benzyltoluene in a trickle-bed reactor – influence of mass transfer and optimization strategies. Int. J. Hydrogen Energy.

[cit73] García A., Marín P., Ordóñez S. (2024). Hydrogenation of liquid organic hydrogen carriers: Process scale-up, economic analysis and optimization. Int. J. Hydrogen Energy.

[cit74] Le T. H., Tran N., Lee H. J. (2024). Development of Liquid Organic Hydrogen Carriers for Hydrogen Storage and Transport. Int. J. Mol. Sci..

[cit75] Wang J. (2025). *et al.*, Modelling and kinetics of the toluene/methylcyclohexane-based hydrogen storage system. Can. J. Chem. Eng..

[cit76] Roy S., Newalkar B. L., Datta S. (2014). Vanadium substituted SBA-15 supported bimetallic Pt, Pd catalysts for hydrogenation of toluene to methylcyclohexane. Can. J. Chem. Eng..

[cit77] Prechtl M. H., Scholten J. D., Dupont J. (2009). Tuning the selectivity of ruthenium nanoscale catalysts with functionalised ionic liquids: Hydrogenation of nitriles. J. Mol. Catal. A: Chem..

[cit78] Wang J. (2025). *et al.*, Modelling and kinetics of the toluene/methylcyclohexane-based hydrogen storage system. Can. J. Chem. Eng..

[cit79] Jorschick H. (2017). *et al.*, Hydrogen storage using a hot pressure swing reactor. Energy Environ. Sci..

[cit80] Li Y. (2024). *et al.*, Boosting dehydrogenation of dodecahydro-N-ethylcarbazole over Pd nanoclusters with tailored electronic structures loaded on nitrogen-doped carbon. Int. J. Hydrogen Energy.

[cit81] Dong Y. (2016). *et al.*, Dehydrogenation kinetics study of perhydro-N-ethylcarbazole over a supported Pd catalyst for hydrogen storage application. Int. J. Hydrogen Energy.

[cit82] Yasin Q. U. A. (2026). *et al.*, Liquid organic hydrogen carriers for hydrogen storage: A review of chemistry, engineering design and economic feasibility. Energy Convers. Manage.:X.

[cit83] Ramadhani S. (2024). *et al.*, Advances in Catalytic Hydrogenation of Liquid Organic Hydrogen Carriers (LOHCs) Using High-Purity and Low-Purity Hydrogen. ChemCatChem.

[cit84] Zhang K. (2025). *et al.*, Recent advances in dimethyl oxalate hydrogenation: integrating catalyst design with reaction engineering for sustainable production of C2oxygenates. J. Mater. Chem. A.

[cit85] Wen X. (2026). *et al.*, Safety-oriented catalytic hydrogenation based on supported catalysts: research progress and perspectives. J. Loss Prev. Process Ind..

[cit86] McNair R. J. , The process of catalyst selection, in Catalysis of Organic Reactions. 2017. p. 1–11

[cit87] Gong W. (2024). *et al.*, Unlocking the catalytic potential of heterogeneous nonprecious metals for selective hydrogenation reactions. Chem. Soc. Rev..

[cit88] Zhang J. (2021). *et al.*, Single-atom catalysts for thermal- and electro-catalytic hydrogenation reactions. J. Mater. Chem. A.

[cit89] Xu M. (2024). *et al.*, Study on the dehydrogenation of perhydro-dibenzyltoluene catalyzed by Pt/Al2O3 in a fixed bed reactor. Chem. Eng. Sci..

[cit90] Ali A., Rohini A. K., Lee H. J. (2022). Dehydrogenation of perhydro-dibenzyltoluene for hydrogen production in a microchannel reactor. Int. J. Hydrogen Energy.

[cit91] Schörner M. (2024). *et al.*, Inductively heatable catalytic materials for the dehydrogenation of the liquid organic hydrogen carrier (LOHC) perhydro dibenzyltoluene. Catal. Sci. Technol..

[cit92] Geißelbrecht M. (2024). *et al.*, Modeling of the Continuous Dehydrogenation of Perhydro-Dibenzyltoluene in a Cuboid Reactor. Energy Technol..

[cit93] Yang Y. (2023). *et al.*, Review on the thermal neutrality of application-oriented liquid organic hydrogen carrier for hydrogen energy storage and delivery. Results Eng..

[cit94] Ahn C.-I. (2022). *et al.*, Dehydrogenation of homocyclic liquid organic hydrogen carriers (LOHCs) over Pt supported on an ordered pore structure of 3-D cubic mesoporous KIT-6 silica. Appl. Catal., B.

[cit95] Niermann M. (2019). *et al.*, Liquid Organic Hydrogen Carrier (LOHC)–Assessment based on chemical and economic properties. Int. J. Hydrogen Energy.

[cit96] Bollmann J. (2023). *et al.*, Burner-heated dehydrogenation of a liquid organic hydrogen carrier (LOHC) system. Int. J. Hydrogen Energy.

[cit97] Faye O., Szpunar J., Eduok U. (2022). A critical review on the current technologies for the generation, storage, and transportation of hydrogen. Int. J. Hydrogen Energy.

[cit98] Markiewicz M. (2019). *et al.*, Hazard assessment of quinaldine-, alkylcarbazole-, benzene-and toluene-based liquid organic hydrogen carrier (LOHCs) systems. Energy Environ. Sci..

[cit99] Li H. (2014). *et al.*, Progresses in hydrogenation or dehydrogenation of naphthalene, tetralin and decalin, and the catalysts. Shiyou Huagong.

[cit100] Nuzhdin A. L., Bukhtiyarova M. V., Bukhtiyarova G. A. (2024). Organic synthesis in flow mode by selective liquid-phase hydrogenation over heterogeneous non-noble metal catalysts. Org. Biomol. Chem..

[cit101] Zhu M. (2018). *et al.*, Palladium supported on carbon nanotubes as a high-performance catalyst for the dehydrogenation of dodecahydro-N-ethylcarbazole. Catalysts.

[cit102] Amende M. (2014). *et al.*, Model Catalytic Studies of Liquid Organic Hydrogen Carriers: Dehydrogenation and Decomposition Mechanisms of Dodecahydro-N-ethylcarbazole on Pt(111). ACS Catal..

[cit103] Cabrera G. (2024). Liquid Organic Hydrogen Carrier Concepts and Catalysts: Progress and Prospects. Molecules.

[cit104] Jensen C. , et al., Development of a Practical Hydrogen Storage System Based on Liquid Organic Hydrogen Carriers and a Homogeneous Catalyst, Hawaii Hydrogen Carriers, LLC, Honolulu, HI (United States). 2017

[cit105] Zhou M. J. (2024). *et al.*, Recent advances in reversible liquid organic hydrogen carrier systems: From hydrogen carriers to catalysts. Adv. Mater..

[cit106] Cabrera G. (2024). *et al.*, Liquid Organic Hydrogen Carrier Concepts and Catalysts for Hydrogenation and Dehydrogenation Reactions. Molecules.

[cit107] Wen X. (2026). *et al.*, Safety-oriented catalytic hydrogenation based on supported catalysts: research progress and perspectives. J. Loss Prev. Process Ind..

[cit108] Toppinen S. (1996). *et al.*, Kinetics of the liquid-phase hydrogenation of benzene and some monosubstituted alkylbenzenes over a nickel catalyst. Ind. Eng. Chem. Res..

[cit109] Barrio V. (2003). *et al.*, Aromatics hydrogenation on silica–alumina supported palladium–nickel catalysts. Appl. Catal., A.

[cit110] He T., Pei Q., Chen P. (2015). Liquid organic hydrogen carriers. J. Energy Chem..

[cit111] Rautanen P. A. (2002). *et al.*, Liquid-phase hydrogenation of naphthalene and tetralin on Ni/Al2O3: Kinetic modeling. Ind. Eng. Chem. Res..

[cit112] Kirumakki S. R. (2006). *et al.*, Hydrogenation of naphthalene over NiO/SiO2–Al2O3 catalysts: structure–activity correlation. J. Catal..

[cit113] Sakanishi K. (1989). *et al.*, The reactivities of polyaromatic hydrocarbons in catalytic hydrogenation over supported noble metals. Bull. Chem. Soc. Jpn..

[cit114] Sun B. (2013). *et al.*, NanoRu@ hectorite: a heterogeneous catalyst with switchable selectivity for the hydrogenation of quinoline. Appl. Catal., A.

[cit115] Bianchini C. , Meli A., and Vizza F., Hydrogenation of arenes and heteroatoms, in Handbook of Homogeneous Hydrogenation, Wiley-VCH Weinheim, Germany. 2007

[cit116] Aakko-Saksa P. T. (2018). *et al.*, Liquid organic hydrogen carriers for transportation and storing of renewable energy–Review and discussion. J. Power Sources.

[cit117] Stalzer M. M. (2016). *et al.*, Single-Face/All-cis Arene Hydrogenation by a Supported Single-Site d0 Organozirconium Catalyst. Angew. Chem..

[cit118] Fareed B. (2025). *et al.*, Advanced monometallic and bimetallic catalysts for energy efficient propylene production via propane dehydrogenation pathways–A review. Appl. Energy.

[cit119] Sotoodeh F., Huber B. J., Smith K. J. (2012). Dehydrogenation kinetics and catalysis of organic heteroaromatics for hydrogen storage. Int. J. Hydrogen Energy.

[cit120] Orava V., Souček O., Cendula P. (2015). Multi-phase modeling of non-isothermal reactive flow in fluidized bed reactors. J. Comput. Appl. Math..

[cit121] Shi L. (2019). *et al.*, Integration of hydrogenation and dehydrogenation based on dibenzyltoluene as liquid organic hydrogen energy carrier. Int. J. Hydrogen Energy.

[cit122] Modisha P., Bessarabov D. (2020). Stress tolerance assessment of dibenzyltoluene-based liquid organic hydrogen carriers. Sustainable Energy Fuels.

[cit123] Bulgarin A. (2020). *et al.*, Purity of hydrogen released from the Liquid Organic Hydrogen Carrier compound perhydro dibenzyltoluene by catalytic dehydrogenation. Int. J. Hydrogen Energy.

[cit124] Dai M. (2025). *et al.*, A review of reversible hydrogenation and dehydrogenation catalysts for liquid organic hydrogen carriers. Catal. Sci. Technol..

[cit125] Aditiya H. B. (2025). *et al.*, Hydrogen transport across oceans: A techno-economic assessment of hydrogen carriers. Appl. Energy.

[cit126] Yasin Q. U. A. (2026). *et al.*, Liquid organic hydrogen carriers for hydrogen storage: A review of chemistry, engineering design and economic feasibility. Energy Convers. Manage.:X.

[cit127] Ahn B., Liu J. J., Won W. (2023). Process development for efficient hydrogen transportation and analyses: technical, economic, and environmental perspectives. Comput.-Aided Chem. Eng..

[cit128] Shyong J. (2026). *et al.*, Technoeconomic analysis of hydrogen storage using 1,4-butanediol (BDO)/γ-butyrolactone (GBL) as a stationary backup power system. Int. J. Hydrogen Energy.

[cit129] Uijthof E. M. T. (2024). *et al.*, Liquid organic hydrogen carriers: Process design and economic analysis for manufacturing N-ethylcarbazole. J. Adv. Manuf. Process..

[cit130] Byun M. (2022). *et al.*, Preliminary feasibility study for hydrogen storage using several promising liquid organic hydrogen carriers: Technical, economic, and environmental perspectives. Energy Convers. Manage..

[cit131] Distel M. M. (2025). *et al.*, Large-Scale H2 Storage and Transport with Liquid Organic Hydrogen Carrier Technology: Insights into Current Project Developments and the Future Outlook. Energy Technol..

[cit132] Bontron C. , *et al.*, Life cycle assessment of two liquid organic hydrogen carriers, in Computer Aided Chemical Engineering, Elsevier. 2023. p. 3381–3386

[cit133] Cho H. H., Strezov V., Evans T. J. (2024). Life cycle assessment of renewable hydrogen transport by liquid organic hydrogen carriers. J. Cleaner Prod..

[cit134] International Energy Agency (IEA) , Ammonia Technology Roadmap: towards More Sustainable Nitrogen Fertiliser Production, OECD Publishing, Paris. 2021, 10.1787/f6daa4a0-en

[cit135] Aldilaijan R. (2025). *et al.*, Kinetic modelling and process optimization for low-carbon hydrogen production via ammonia cracking. Int. J. Hydrogen Energy.

[cit136] van der Laag R. (2026). *et al.*, Machine learning for hydrogen technologies: A comprehensive review of challenges, opportunities, and emerging trends. Int. J. Hydrogen Energy.

[cit137] Lin C. (2025). *et al.*, Harnessing physics-inspired machine learning to design nanocluster catalysts for dehydrogenating liquid organic hydrogen carriers. Appl. Catal., B.

[cit138] Li H. (2024). *et al.*, Application and Analysis of Liquid Organic Hydrogen Carrier (LOHC) Technology in Practical Projects. Energies.

[cit139] Aditya D. S. (2025). *et al.*, A sustainable approach to lithium and industrial wastewater separation using recycled cellulose membrane with Zr-functionalized silicate clay. Chem. Eng. J..

[cit140] Chilunda M. D. (2025). *et al.*, Electrochemical cycling of liquid organic hydrogen carriers as a sustainable approach for hydrogen storage and transportation. ACS Sustain. Chem. Eng..

[cit141] Lebedeva O. (2023). *et al.*, Advances and prospects in electrocatalytic hydrogenation of aromatic hydrocarbons for synthesis of “loaded” liquid organic hydrogen carriers. Curr. Opin. Electrochem..

[cit142] Craig V. S. , Gas solubility of electrolytes, Encyclopedia of Applied Electrochemistry, 2014, pp. 927–931

[cit143] Lebedeva O., Kultin D. Y., Kustov L. (2021). Electrochemical Behavior of Benzene, Diphenyl, and p-Terphenyl in Room-Temperature Ionic Liquid N-Butylpyridinium Chloride–AlCl3. Russ. J. Phys. Chem. A.

[cit144] Lee C. J. (2025). *et al.*, An efficient toluene barrier membrane for high-performance direct toluene hydrogenation via an electrochemical process. J. Mater. Chem. A.

[cit145] Sievi G. (2019). *et al.*, Towards an efficient liquid organic hydrogen carrier fuel cell concept. Energy Environ. Sci..

[cit146] Cho J. (2023). *et al.*, Electrochemically activatable liquid organic hydrogen carriers and their applications. J. Am. Chem. Soc..

[cit147] Sampson J. , Intelligent Energy signs MoU to develop China fuel cell automotive market, Available at: https://www.h2-view.com/story/intelligent-energy-signs-mou-to-develop-china-fuel-cell-automotive-market/.H2View, 2019

[cit148] Plant M. , Green hydrogen plans for German region in GET H2 initiative Fuel Cells Bull, pp. 11–12, 2019, 10.1016/s1464-2859(19)30205-6

[cit149] Al K., Kantürk Figen A. (2025). Dibenzyltoluene-based liquid organic hydrogen carrier systems: Recent advances, challenges, and future perspectives. Clean Energy Technol. J..

[cit150] Bermudez Aponte N. A., Meille V. (2024). Use of biosourced molecules as liquid organic hydrogen carriers (LOHC) and for circular storage. Reactions.

[cit151] Huang Y. (2023). *et al.*, Integration of Hydrogenation and Dehydrogenation Based on N-Ethylcarbazole as a Liquid Organic Hydrogen Carrier. Ind. Eng. Chem. Res..

[cit152] Liu H. (2023). *et al.*, Hydrogenation of N-ethylcarbazole with hydrogen-methane mixtures for hydrogen storage. Fuel.

